# Species Distribution Modeling Reveals Future Climate Refugia and Important Areas for Rocky Plants in Brazil's Iron Quadrangle

**DOI:** 10.1002/ece3.73355

**Published:** 2026-04-05

**Authors:** Ana Flávia Francisconi, João Victor da Silva Rabelo‐Araujo, Ana Cristina Silva Amoroso Anastacio, Ana Paula da Silva Marques, Maria Imaculada Zucchi

**Affiliations:** ^1^ Escola Superior de Agricultura “Luiz de Queiroz” Universidade de São Paulo Piracicaba Brazil; ^2^ Instituto de Biologia Universidade Estadual de Campinas Campinas Brazil; ^3^ Vale S.A. Nova Lima Brazil; ^4^ Secretaria de Agricultura e Abastecimento do Estado de São Paulo, APTA‐URPD Piracicaba Brazil

**Keywords:** climate change, climate refugia, conservation planning, endemism, microendemism, rupestrian grasslands

## Abstract

Climate change is expected to substantially alter the distribution of rupestrian plant species in the Iron Quadrangle (IQ), southeastern Brazil, with important implications for conservation planning. In this study, we assessed how future climate scenarios may affect the distribution of eight rupestrian angiosperms, *Aiouea tetragona* (Lauraceae), *Dyckia consimilis*, *D. rariflora*, *Hoplocryptanthus ferrarius*, *H. schwackeanus*, *Paepalanthus amoenus*, *P. magalhaesii* (Eriocaulaceae), and *Vriesea minarum* (Bromeliaceae), and identified priority areas for conservation. Occurrence records were compiled from online herbaria and datasets provided by Vale S.A., followed by spatial thinning to reduce sampling bias. Ensemble ecological niche models were fitted using ENMTML based on current climatic suitability derived from WorldClim v2.1 bioclimatic variables. Future projections incorporated three CMIP6 general circulation models under SSP1‐2.6 and SSP5‐8.5 scenarios for 2050 and 2090. We further quantified the overlap between climatically suitable areas and protected areas, identifying candidate climatic refugia and potential ecological corridors. All species achieved acceptable to excellent predictive performance (AUC > 0.75; TSS > 0.5). Projections indicated widespread contractions in climatically suitable areas, particularly for xeromorphic taxa such as *A. tetragona*, *D. rariflora*, *H. ferrarius*, and *P. magalhaesii*, which approached near‐zero suitability by 2090 under SSP5‐8.5. In contrast, *D. consimilis* and *H. schwackeanus* showed comparatively stable suitability patterns, while *P. amoenus* and *V. minarum* exhibited transient gains under intermediate scenarios. Persistent suitability hotspots were concentrated along the Gandarela–Caraça axis, including areas such as Serra do Gandarela and the RPPN Horto Alegria, indicating climatically stable zones that may function as future refugia for edaphically specialized rocky‐field species. Our results indicate that climate mitigation alone will likely be insufficient to safeguard rupestrian flora in the Iron Quadrangle. Proactive conservation strategies will be necessary, including strengthening ecological connectivity, establishing seed banks and germplasm collections, and planning assisted translocations. Additionally, we provide an interactive tool designed to translate model outputs into management‐oriented products that can support decision‐making and prioritize conservation interventions in key landscapes such as the Gandarela–Caraça corridor.

## Graphical abstract

Climate change is projected to sharply reduce suitable habitats for eight endemic rupestrian plant species in Brazil's Iron Quadrangle. Ensemble niche models reveal species‐specific vulnerabilities and highlight climatic refugia and corridors that may support persistence. These results provide a spatial framework to guide conservation and restoration planning in a rapidly changing landscape.

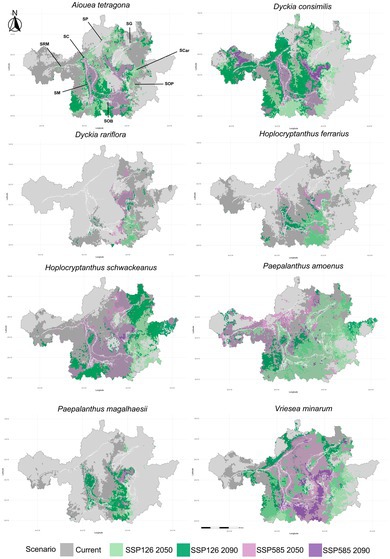

## 1 Introduction

Ongoing anthropogenic climate change is among the most significant drivers of shifts in species distributions at global and regional scales during the 21st century, primarily through alterations in temperature and precipitation regimes (Johnston et al. 2024). These climatic changes can modify the spatial configuration of environmentally suitable areas, particularly for species with narrow ecological requirements (Rota et al. 2022; Cai et al. 2022).

The geographic distribution of rare and endemic plant species is strongly shaped by adaptation to unique and often restrictive edaphic conditions, such as iron‐rich soils or wetlands, providing specialized habitats essential for their survival (Negreiros et al. 2014; Li et al. 2024; Silveira et al. 2016). In many narrow endemics, limited seed dispersal and reduced gene flow have been reported and may contribute to low genetic diversity and elevated levels of intraspecific inbreeding within small and spatially isolated populations (Rego et al. 2025). These intrinsic constraints, when combined with human‐driven habitat fragmentation, land‐use change, and mineral resource extraction, are widely recognized as factors that can increase vulnerability to rapid environmental change (Brabant et al. 2025; Keck et al. 2025).

These vulnerabilities are particularly pronounced in the Iron Quadrangle (IQ), one of the world's largest iron ore reserves in southeastern Brazil. This geologically unique region harbors a remarkable concentration of endemic plant species adapted to ferruginous and quartzitic substrates (Jacobi and do Carmo 2008; Paraguassú et al. 2019).

The IQ comprises a mosaic of ancient landscapes and extreme edaphic conditions that support specialized habitats, including the lithobiome of rupestrian grasslands, locally known as *campos rupestres* (from Portuguese: *campos* = fields; *rupestres* = rocky), characterized by open, rocky montane vegetation growing on nutrient‐poor substrates. Nevertheless, these very attributes confer a heightened susceptibility to ecological disturbance, predominantly as a consequence of intensive mining activities, alterations in land use, urban expansion, and the incidence of both natural and anthropogenic fires (Azevedo et al. 2024; Spier et al. 2003).

Predictive macroclimatic distribution models indicate that rupestrian grasslands in the Espinhaço Mountain Range may lose up to 95% of their climatically suitable area by the end of the century under future warming scenarios (Fernandes et al. 2014). Despite extensive anthropogenic alterations to the landscape, the Iron Quadrangle (IQ) may retain relatively higher climatic stability compared to northern portions of the Espinhaço Range. Its pronounced altitudinal gradients, complex topography, and associated climatic heterogeneity may favor the persistence of localized microclimatic refugia, potentially allowing the region to function as an isolated climatic refuge for rupestrian grasslands and their biodiversity under future climate scenarios (Salles et al. 2019).

Although the region hosts numerous protected areas—such as environmental protection areas (APAs), private natural heritage reserves (RPPNs), and public parks—several species within these areas remain poorly studied, and comprehensive strategies for their long‐term in situ and *ex situ* conservation are still in the early stages (Lavor et al. 2014; Lemes et al. 2021). This gap is particularly concerning given the urgent need for robust predictive tools to assess the impacts of climate change on biodiversity persistence within the IQ, thereby informing effective and proactive conservation planning (Cavalcanti et al. 2023; Jacobi et al. 2011).

Species distribution models (SDMs) are a vital tool for understanding the relationship between species occurrence records and environmental variables. It enables the estimation of potential habitat suitability across spatial and temporal scales, as well as the prediction of species' responses to environmental changes (Beery et al. 2021).

Although SDMs are widely recognized as valuable tools for predicting habitat suitability, their effective integration into conservation planning, particularly for endemic and microendemic species such as those in the IQ, is frequently limited by the challenge of translating model outputs into clear and actionable guidance for decision‐makers (Muscatello et al. 2021; Qazi et al. 2022; Sofaer et al. 2019). Meeting the conservation needs of narrowly distributed species therefore requires not only robust environmental niche modeling but also careful consideration of the unique ecological and landscape complexities of the IQ, including species‐specific reproductive biology and dispersal strategies (Lee‐Yaw et al. 2022; Villero et al. 2017).

Building upon this context, the present study models the current and future distributions of eight rupestrian species endemic or restricted to the IQ, each characterized by distinct ecological and reproductive adaptations to the region's unique edaphic conditions. These species include the shrub *Aiouea tetragona*; the saxicolous bromeliads *Dyckia consimilis* and *D*. *rariflora*; the closely related rupicolous species *Hoplocryptanthus ferrarius* and *H*. *schwackeanus*; the widely distributed tank bromeliad *Vriesea minarum*; and two primarily anemochorous Eriocaulaceae species, *Paepalanthus amoenus* and *P*. *magalhaesii*. The selected species encompass contrasting life forms, reproductive and dispersal strategies, substrate affinities, and degrees of geographic restriction within the IQ. This ecological variation provides a structured framework to compare projected climate‐driven shifts across distinct functional and distributional profiles.

Accordingly, this study aims to (i) model the potential distributions of these species under current climatic conditions, (ii) evaluate projected changes in climatically suitable areas under future climate scenarios, and (iii) apply these insights to inform targeted in situ conservation strategies, including identification of priority areas for protection and guidance for potential translocation efforts within the Iron Quadrangle.

## 2 Material and Methods

### 2.1 Study Area

**FIGURE 1:**
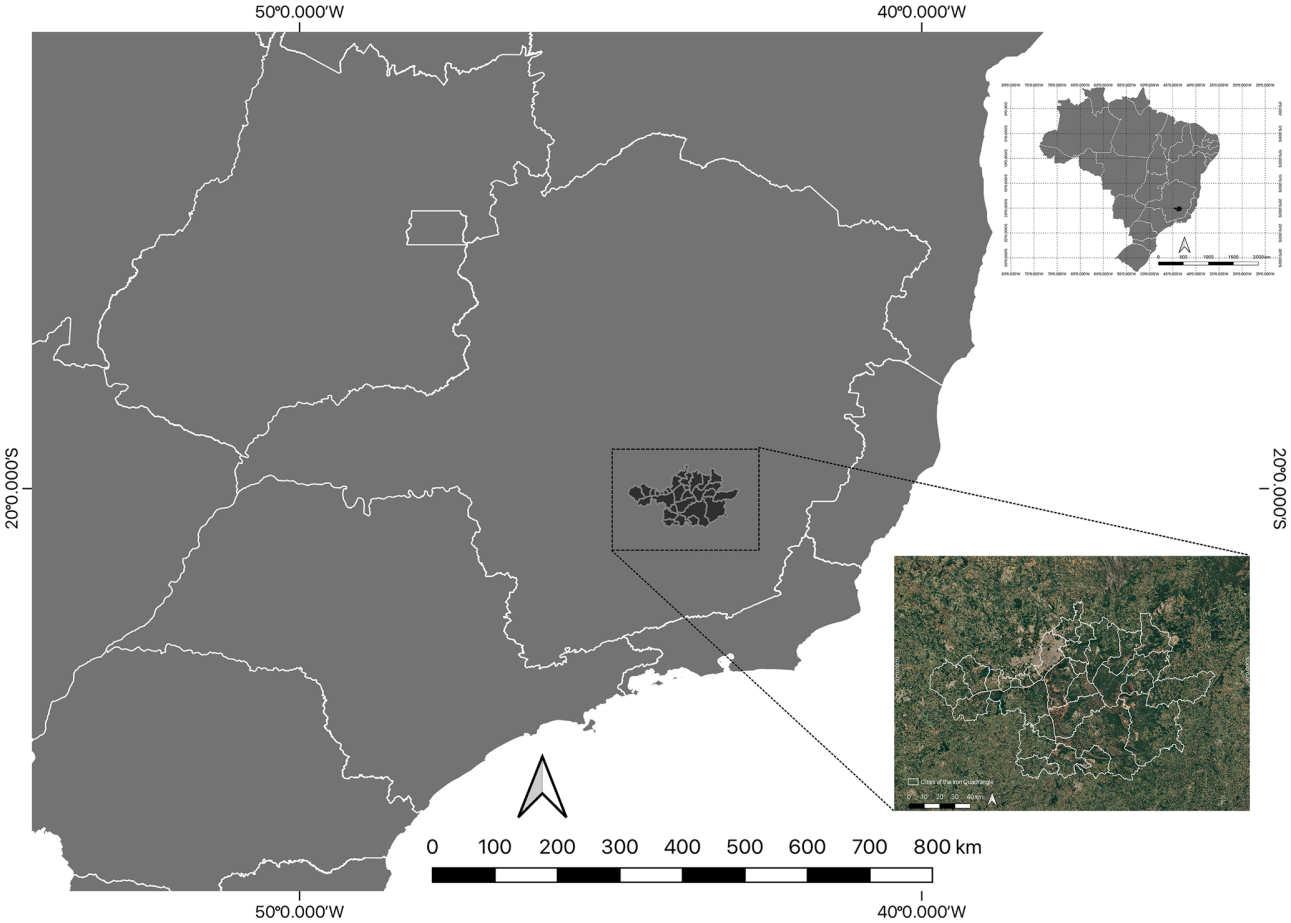
Location of the Iron Quadrangle (IQ) in Minas Gerais, Brazil. The small map shows the position of the IQ within the country, while the larger map highlights the municipalities that compose the IQ. The shapefile of the area was obtained from the Prístino Institute repository (https://institutopristino.org.br/atlas/quadrilatero‐ferrifero/). All maps are shown in UTM projection (zone 23S, WGS84 datum).

The IQ, situated in the south‐central region of the state of Minas Gerais, represents a geological formation composed of ancient tonalitic‐granitic gneisses from the Precambrian, which are roughly arranged in the shape of an apparent quadrangle characterized by extensive synclines (Figure 1) (de Vicq Ferreira da Costa et al. 2025). Spanning an area of approximately 7000 km^2^, the IQ is one of the largest and most significant mineral provinces in Brazil, internationally recognized as one of the world's extensive reserves of mineral resources, such as iron, gold, and manganese (Spier et al. 2007).

The mesoclimate of the mountainous region fluctuates with altitude variation, which ranges between 700 and 2000 m above sea level. It is primarily characterized as semi‐humid tropical, with precipitation mainly occurring from October to March. This climate facilitates the presence of semi‐deciduous forests, grassland‐woody savannas, and, at higher plateaus, open “Campo Rupestre” fields (Salgado and Fonseca do Carmo 2015).

### 2.2 Analyzed Species

**FIGURE 2:**
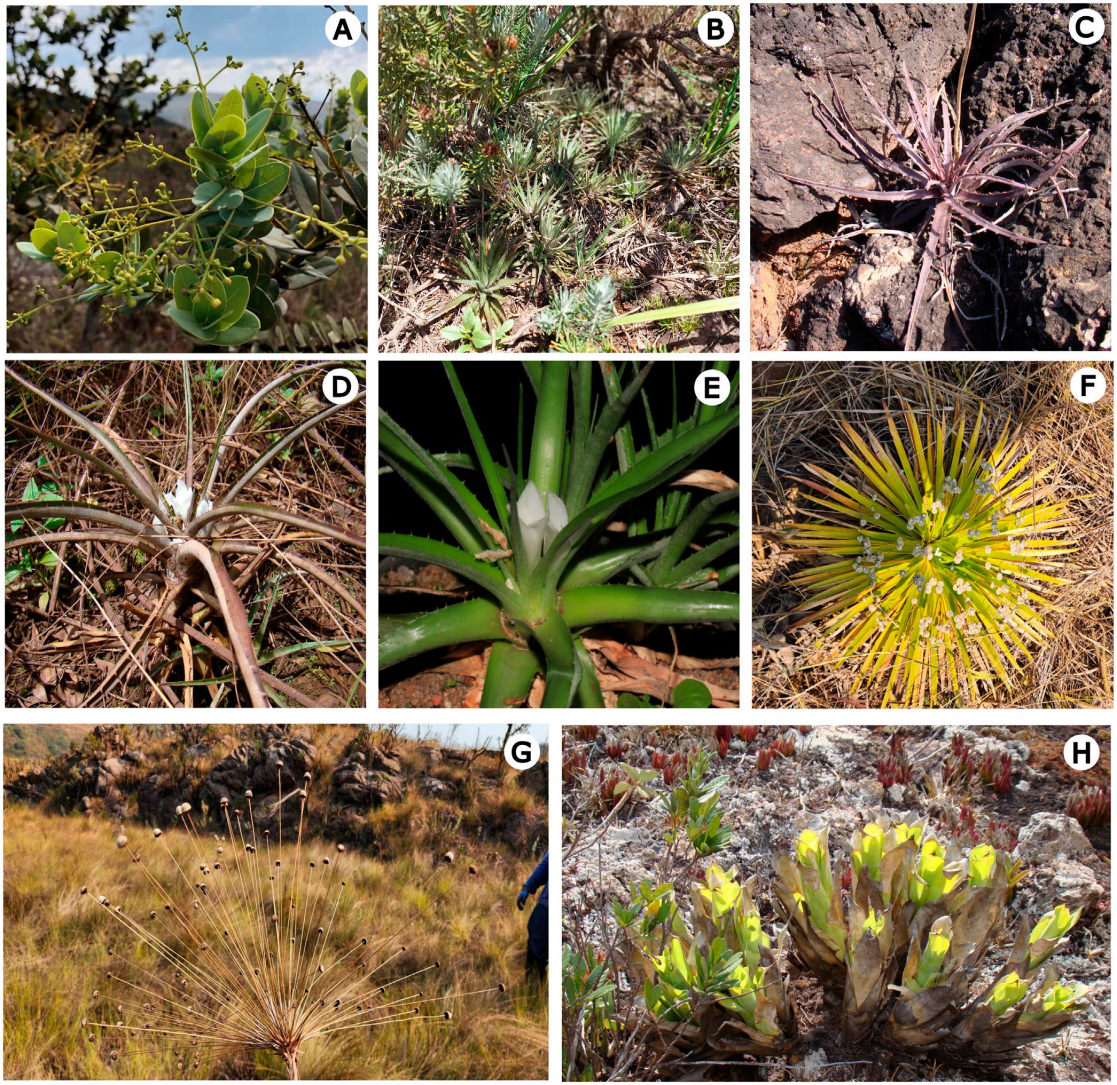
Representative species from the Iron Quadrangle included in SDM analyses, illustrating their morphological diversity: (A) *Aiouea tetragona*, (B) *Dyckia consimilis*, (C) *D. rariflora*, (D) *Hoplocryptanthus ferrarius*, (E) *H*. *schwackeanus*, (F) *Paepalanthus amoenus*, (G) *P. magalhaesii*, and (H) *Vriesea minarum*. Photos C, E, and H courtesy of O.B.C. Ribeiro.

To assess the potential impacts of climate change on species' niche distributions in the Campos Rupestres, we selected eight species occurring in the IQ that represent contrasting ecological strategies, life forms, and habitat associations within this ecosystem (Figure 2). These taxa differ in their degrees of geographic restriction, reproductive and dispersal strategies, and substrate associations, providing a comparative basis to evaluate how projected climate‐driven suitability shifts may affect species with distinct ecological and distributional characteristics.

*Aiouea tetragona* (Mez) Rohwer & R. Rohde (Lauraceae) is a medium‐sized shrub with simple, alternate leaves, occurring on substrates under high solar exposure and shallow rocky soils in the IQ. It produces small, whitish flowers arranged in panicles, with tetrasporangiate stamens and tepals in two whorls, a typical feature of Lauraceae (Rohde et al. 2017).

*Dyckia consimilis* Mez (Bromeliaceae) is a saxicolous species, with occurrences mostly restricted to elevations between 1,336 and 1,600 m along the Sinclinal Moeda in the western IQ. It propagates clonally through a guerrilla strategy, forming small rosettes and compact population clusters (Guarçoni et al. 2010).

*Dyckia rariflora* Schult. & Schult.f. (Bromeliaceae) has a wider distribution across the eastern IQ, including the Serra de Ouro Preto, Serra de Antônio Pereira, and Serra do Caraça, at elevations from 806 to 1,624 m. It is a solitary, seed‐reproducing rosette pollinated by hummingbirds, bees, and butterflies (da Silva et al. 2024; Pinangé et al. 2017).

*Hoplocryptanthus ferrarius* Leme & C.C. Paula (Bromeliaceae) is narrowly endemic to rocky grasslands and distinguished morphologically by longer leaves relative to *H. schwackeanus*. It commonly co‐occurs with *D. rariflora* and other bromeliads and Velloziaceae (Leme and De Paula 2009). *Hoplocryptanthus schwackeanus* Mez (Bromeliaceae) occurs in shaded, saxicolous or terrestrial habitats, recorded at elevations up to 1,600 m, particularly in the Serra da Piedade (Leme 2007).

*Paepalanthus amoenus* Bong. (Eriocaulaceae) is broadly distributed across quartzitic and ferruginous substrates in the IQ. It is predominantly anemochorous and forms extensive, often disjunct populations (Trovó and Sano 2011).

*Paepalanthus magalhaesii* Silveira (Eriocaulaceae) is found mainly in the QF on quartzitic substrates. It is highly fire‐tolerant, an adaptation to frequent natural and anthropogenic fires (Andrino et al. 2022).

*Vriesea minarum* L.B.Sm (Bromeliaceae) is a tank bromeliad that occurs at elevations from 800 to 1,800 m elevation. It is pollinated by hummingbirds and beetles, but its small, fragmented populations are severely threatened by mining, fire, and urban encroachment (Lavor et al. 2014; Versieux 2011).

### 2.3 Data Collection and Filtering

The rupestrian species had their geographical coordinates compiled from online herbaria. To begin the search, we defined the scientific names and synonyms of the species (Table S1), based on information available in Reflora (https://floradobrasil.jbrj.gov.br/reflora/PrincipalUC/PrincipalUC.do?lingua=pt). Online herbaria were searched using spocc 1.2.3 (https://github.com/ropensci/spocc) and bien 1.2.6 (Maitner et al. 2018) R packages, which returned coordinates from GBIF (https://www.gbif.org/), New York Botanical Garden (https://www.nybg.org/) and specieslink (https://specieslink.net/). In addition to the online herbarium search, coordinates obtained in the field by the Vale S.A. and validated by taxonomists were made available.

After obtaining the data, initial filtering of the coordinates was carried out with the CoordinateCleaner 3.0.1 R package (Zizka et al. 2019) using the following criteria: (i) missing data filter—excluded occurrences that did not contain longitude and/or latitude information and those that were duplicates or zeros; (ii) spatial filter—considered only coordinates that were not located in capital cities (2,000 m), centroids of countries or municipalities (2,000 m), GBIF headquarters, biodiversity institutions (100 m), ocean and within urban areas; (iii) testing for outliers, the quantile method was used, and five was the number of data points among all occurrences to identify an outlier; (iv) filtering out possible nomenclature and geolocation errors.

The most common issue affecting species data in online herbaria was duplicate records, followed by entries containing zeros. Duplicate records were removed prior to modeling by retaining only one occurrence per identical latitude–longitude pair. When multiple records shared the same coordinates across different databases, a single representative entry was maintained to avoid spatial redundancy and artificial inflation of sampling effort. Additionally, some access records lacked coordinate information. A final filtering step was performed to consider only the coordinates within the IQ. Using the sf 1.0.19 R package (Pebesma 2018) points were identified and extracted based on their intersection with the shapefile boundaries, ensuring that only coordinates within the target area of interest were selected.

In addition to automated filtering with CoordinateCleaner, all remaining occurrences were visually inspected to confirm their consistency with the known distribution of each species within the IQ and to identify potential georeferencing errors. For each species, between 12 and 55 geographic coordinates were used (Tables S2 and S3).

### 2.4 Current and Future Climatic Data

To model the species of the IQ, we used 19 bioclimatic variables (bio1 to bio19) from WorldClim 2.1 (1970–2000) at a resolution of 30 arc‐seconds. According to WorldClim, bioclimatic variables represent annual trends (e.g., average annual temperature, annual precipitation), seasonality (e.g., annual amplitude of temperature and precipitation), and extreme or limiting environmental factors (e.g., temperature of the coldest and hottest month, and precipitation of the wet and dry quarters). To project the potential future distribution, we used global climate models from the Coupled Model Intercomparison Project Phase 6 (CMIP6), the most recent generation of climate projections, specifically EC‐EARTH3‐veg, INM‐CM5‐0, and MRI‐ESM2‐0. These models were selected because Bazzanela et al. (2024) identified them as the best performing for both the Northeast and Southeast regions of Brazil, which converge in the IQ, a climatic transition zone characterized by strong altitudinal gradients. Their demonstrated regional performance increases confidence in projected temperature and precipitation shifts for this topographically complex mining landscape.

Two shared socioeconomic scenarios (SSP) were considered: (1) 1–2.6 (SSP126), which represents an optimistic scenario characterized by a shift toward more sustainable practices and low greenhouse gas concentration levels, and (2) 5–8.5 (SSP585), a pessimistic scenario driven by a fossil‐based economy and increasing greenhouse gas emissions (O'Neill, Oppenheimer, et al. 2017; Riahi et al. 2017). Two time periods were used to predict changes in the future: 2050 (average of 2041 to 2060) and 2090 (2081 to 2100). All variables and projections for the future were delimited to the IQ.

Although edaphic and land‐use variables are ecologically relevant in rupestrian systems, they were not incorporated due to the lack of spatially consistent high‐resolution datasets and the incomplete ecological characterization of several narrow endemic species. Including such predictors could artificially restrict modeled suitability to currently sampled lithological contexts, potentially reinforcing sampling bias. Climatic predictors were therefore used to assess broader macroclimatic suitability patterns.

### 2.5 Ecological Niche Modeling

The coordinates filtered according to the CoordinateCleanear parameters, together with the bioclimatic variables restricted to the IQ area were used in the ENMTML 1.0 R package for ecological niche modeling (ENM) (de Andrade et al. 2020). This package performs a rarefaction of the points, in this case removing any coordinate that is within 2× the size of the cell (approximately 2 km). This step helps mitigate overestimation in locations with many close coordinates.

A principal coordinates analysis (PCA) was carried out on the 19 WorldClim bioclimatic variables using a correlation matrix to reduce multicollinearity. The eigenvectors were used to calculate the scores of derived principal components, which were then used as predictor variables in ENMTML R package (“colin_var = c(method = ‘PCA’)”). Components explaining at least 95% of the total variance were retained. The first four principal components (PC1–PC4), cumulatively explaining 96.8% of the variance (Tables S4 and S5), were used in all models. Because the same environmental predictor set and calibration extent were applied, the PCA solution was identical across species. The same eigenvectors were used to calculate PCs scores for future climate scenarios. The model fit was delimited by a buffer with a width equal to the maximum distance between pairs of occurrences for each species. This was done using the “sp_accessible_area” argument, with the “BUFFER” method.

To carry out the modeling, different algorithms were used to estimate the species' niche. The main groups of algorithms are: (i) presence only, (ii) presence and absence, (iii) presence and background (Guisan et al. 2017). Among these three groups, four algorithms were applied to create the MNEs: (A) Bioclim (Booth et al. 2014) for presence only, (B) Random Forest (Breiman 2001), (C) Support Vector Machine (SVM; Guo et al. 2005) both algorithms for presence and absence, and (D) MaxEnt (MaxEnt 3.4.1) (Warren and Seifert 2011), considered the algorithm for presence and background. All the algorithms and metrics were applied and generated with the ENMTML R package (de Andrade et al. 2020). In cases where one algorithm did not meet these thresholds (e.g., Maxent for *Paepalanthus amoenus* and Bioclim for *P. magalhaesii*), it was excluded from the ensemble.

The “env_const” approach was used to determine pseudo‐absences in the ENMTML R package, which are environmentally restricted to a region with lower fitness values predicted by a Bioclim model (Booth et al. 2014). To produce more accurate models, the data were split into 70% training and 30% testing. The bootstrap method was also applied, and the procedure was repeated 10 times.

To assess the quality of the models, the metrics Area Under the Curve (AUC) (Metz 1986) and True Skill Statistics (TSS) (Allouche et al. 2006) were used. The AUC can be interpreted as the probability of a randomly chosen presence location being classified as superior to a randomly chosen background point (Merow et al. 2013). AUC values were classified as follows: excellent (> 0.90); good (> 0.80 and ≤ 0.90); acceptable (> 0.70 and ≤ 0.80); weak (> 0.60 and ≤ 0.70); and poor (≤ 0.60). TSS values vary between −1 and 1, where negative values close to zero indicate that the model is no different from those generated at random. Models close to 1 indicate good results, while values close to 0.5 assume acceptable results (de Lima Moraes et al. 2020). So, ensembles were created (Araújo and Peterson 2012) based on the niche modeling considering those with AUC greater than 0.75 and TSS greater than 0.5.

Species distribution models were ensembled in the ENMTML R package using the “MEAN” method. Considering the current distribution, the two future scenarios (SSP126 and SSP585), the two projected decades (2050 and 2090), the four algorithms used, and the ten bootstraps performed, a total of 200 models were generated for the species [Ntotal = (4 × 10) + (2 × 2 × 4 × 10) = 200].

The geographical coordinates and suitability maps were plotted using the raster (Elith et al. 2020), ggplot2 3.5.1 (Wickham 2016), sf (Pebesma 2018) and gridExtra 2.3 (Auguie 2017) R packages, with the determination of the global minimum and maximum suitability values between all rasters (current and future scenarios), using the min() and max() functions on the R platform.

### 2.6 Estimation of Current and Future Areas

To assess the current and future distribution of species in the IQ, continuous suitability maps were first converted into binary presence–absence maps using the ENMTML R package (de Andrade et al. 2020). The threshold applied was the one that maximizes the True Skill Statistic (Allouche et al. 2006), implemented through the function thr = c(type = “MAX_TSS”), classifying as presence (Johnston et al. 2024) all values equal to or greater than the cut‐off, and as absence (0) all values below it.

From these binary maps, the area of occurrence (km^2^) of each species was estimated using the raster 3.6.30 R package (Hijmans and Van Etten 2020). To ensure accuracy in the calculations, all rasters—both current and projected—were automatically reprojected to the UTM coordinate system. After reprojection, the presence area was obtained by multiplying the number of cells with value 1 (presence) by the area of each cell in km^2^.

For spatial visualization, the binary rasters were converted into data frames containing geographic coordinates and presence/absence classification. Subsequently, maps were produced using the ggplot2 R package (Wickham 2016), showing current distributions overlaid with each future scenario.

### 2.7 Identification of Priority Areas for Conservation

To assess conservation strategies, we evaluated the overlap between projected species distributions and existing protected areas in the IQ. We considered two main categories of protected areas: strict protection units (federal, state, or municipal parks) and private natural heritage reserves (RPPNs). Species distribution projections were analyzed separately for 2050 (2041–2060) and 2090 (2081–2100). For each time period, binary distribution layers were overlaid with protected areas to generate maps showing the spatial distribution of all modeled species relative to strict protection units and RPPNs.

To further identify conservation priorities, we produced heatmaps of species overlap for SSP585 projections in 2050 and 2090 independently. Binary rasters of all modeled species were first resampled to a common grid using the nearest‐neighbor method (function resample, raster R package) (Hijmans and Van Etten 2020) to ensure identical resolution and extent. The resampled rasters were then summed pixel by pixel (function Reduce) to generate a layer representing the number of species predicted to co‐occur at each location (range: 1–8) for each time period. The resulting species‐overlap surface was converted to a point‐based data frame and plotted with the ggplot2 R package (Wickham 2016).

To facilitate visualization and exploration of the results, we developed an interactive application in R Shiny (Chen et al. 2024), available at https://labgen.shinyapps.io/ENMBrIQ/. The application comprises three main tabs: (i) Distributions & Projections, which displays current and projected distributions for the eight species, as well as plots showing changes in suitable area across scenarios and time periods; (ii) Critical Map by Species, which presents species distributions under SSP585 for 2050 and 2090 overlaid with the two main categories of protected areas (strict protection units and RPPNs), allowing users to select species and visualize spatial overlaps; and (iii) Hotspots Maps, which displays the sum of overlapping binary projections of species for SSP585 in 2050 and 2090, enabling the identification of areas with higher concentration of species and their spatial relationship with conservation units.

## 3 Results

### 3.1 Occurrence Data and ENMs Performance

All algorithms used in the ecological niche modeling showed satisfactory predictive performance (Table 1). AUC values ranged from acceptable (> 0.75) to excellent (> 0.90), while TSS scores were consistently above 0.5, indicating that all ensembles were significantly better than random expectations. Overall, the models provided projections derived from ensemble models combining multiple algorithms, bootstrap replicates, and GCM scenarios of species distributions under current and future climate conditions.

**TABLE 1:** Quality metrics of ecological niche models based on different algorithms and ensemble techniques.

Species	Algorithm	AUC	TSS	AUC (SD)	TSS (SD)
*Aiouea tetragona*	BIO	0.882	0.764	0.063	0.126
	MEA	0.904	0.729	0.038	0.074
	MXS	0.833	0.671	0.059	0.090
	RDF	0.894	0.671	0.036	0.077
	SVM	0.890	0.700	0.021	0.056
*Dyckia consimilis*	BIO	0.900	0.800	0.060	0.119
	MEA	0.951	0.867	0.038	0.098
	MXS	0.938	0.800	0.036	0.090
	RDF	0.949	0.858	0.040	0.097
	SVM	0.943	0.867	0.043	0.081
*Dyckia rariflora*	BIO	0.829	0.657	0.102	0.204
	MEA	0.898	0.743	0.088	0.176
	MXS	0.835	0.657	0.101	0.154
	RDF	0.886	0.714	0.066	0.095
	SVM	0.833	0.629	0.078	0.154
*Hoplocryptanthus ferrarius*	BIO	0.770	0.540	0.142	0.284
	MEA	0.936	0.860	0.074	0.135
	MXS	0.828	0.720	0.113	0.169
	RDF	0.896	0.860	0.095	0.135
	SVM	0.960	0.900	0.053	0.105
*Hoplocryptanthus schwackeanus*	BIO	0.894	0.788	0.052	0.104
	MEA	0.918	0.800	0.024	0.084
	MXS	0.806	0.553	0.038	0.079
	RDF	0.917	0.800	0.035	0.089
	SVM	0.933	0.818	0.025	0.081
*Paepalanthus amoenus*	BIO	0.864	0.727	0.074	0.148
	MEA	0.817	0.609	0.049	0.061
	RDF	0.778	0.545	0.047	0.086
	SVM	0.783	0.555	0.047	0.067
*Paepalanthus magalhaesii*	MEA	0.963	0.900	0.053	0.129
	MXS	0.919	0.825	0.059	0.121
	RDF	1.000	1.000	0.000	0.000
	SVM	0.969	0.900	0.044	0.129
*Vriesea minarum*	BIO	0.838	0.675	0.060	0.121
	MEA	0.942	0.775	0.039	0.104
	MXS	0.818	0.592	0.070	0.127
	RDF	0.971	0.867	0.032	0.098
	SVM	0.917	0.775	0.050	0.079

*Note:* The quality metrics are Area Under the Curve (AUC) and True Skill Statistics (TSS); SD = standard deviation. Algorithm: BIO = Bioclim; MEA = Mean; MXS = Maxent; RDF = Random Forest; SVM = Support vector machine.

### 3.2 Current and Future Distribution Trends Per Species

#### 3.2.1 Aiouea tetragona

**FIGURE 3:**
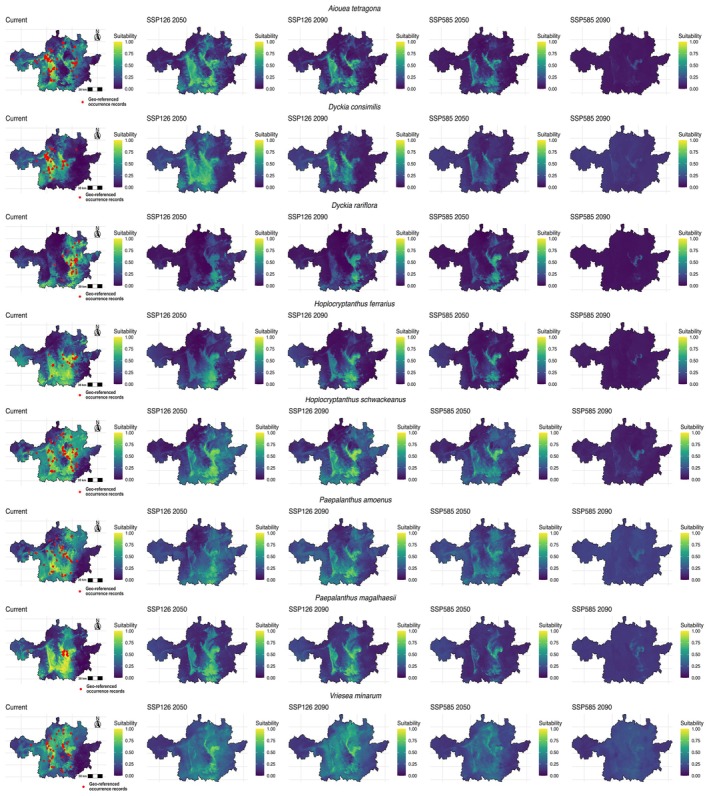
Maps showing the occurrence records, current distribution, and projected future distributions of *A. tetragona* , *D. consimilis* , *D. rariflora*, *H. ferrarius*, *H. schwackeanus*, *P. amoenus* , *P. magalhaesii*, and *V. minarum*. Projections are shown under standardized scales for SSP126, representing an optimistic scenario for 2050 and 2090, and SSP585, a pessimistic scenario for 2050 and 2090. All maps are shown in UTM projection (zone 23S, WGS84 datum).

**FIGURE 4:**
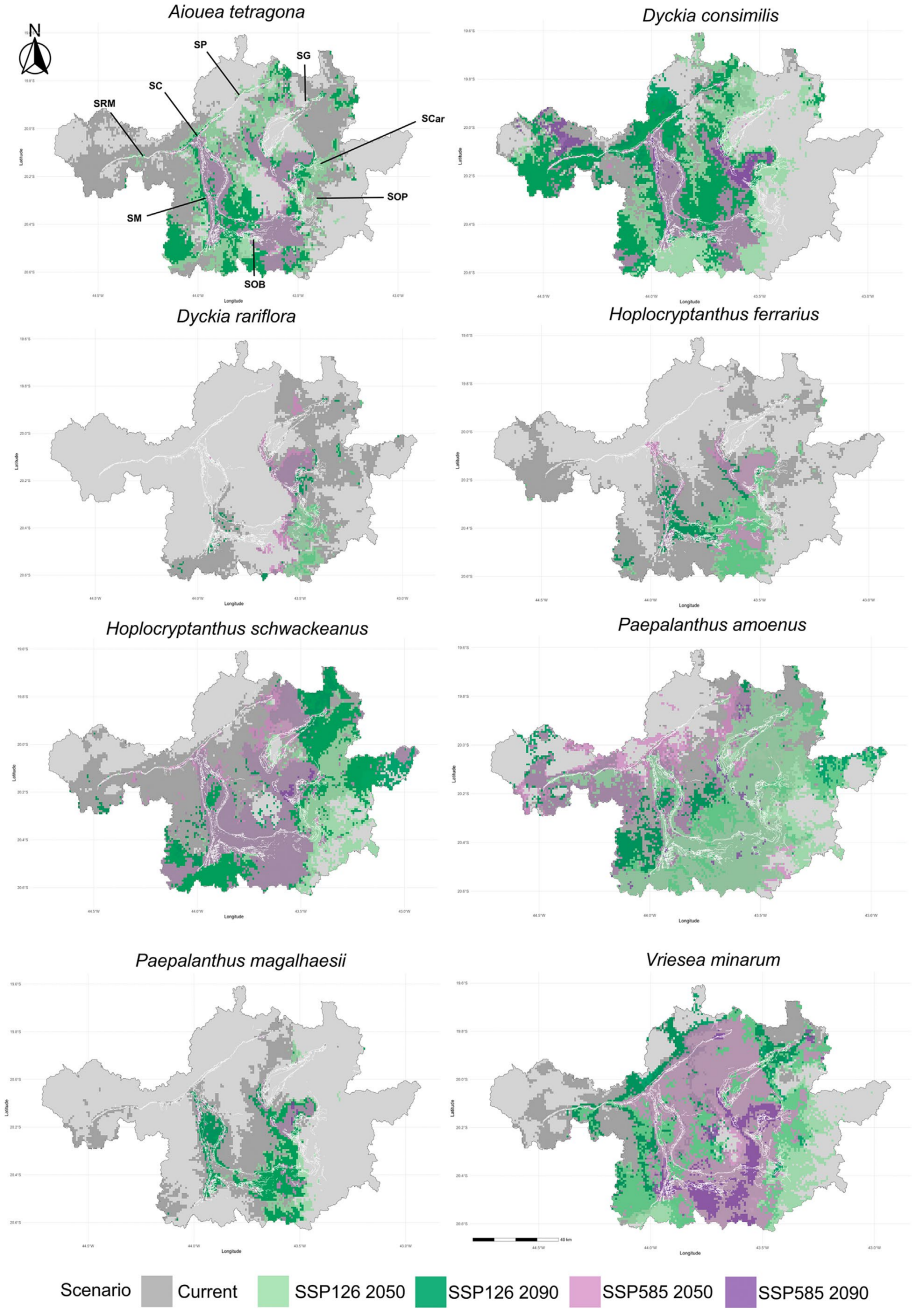
Current and projected distributions of the eight species under optimistic (SSP126 for 2050 and 2090) and pessimistic (SSP585 for 2050 and 2090) climate scenarios, shown as overlaid maps. SC = Serra do Curral; SCar = Serra do Caraça; SG = Serra do Gandarela; SM = Sinclinal Moeda; SOB = Serra de Ouro Branco; SOP = Serra de Ouro Preto; SP = Serra da Piedade; SRM = Serra do Rola Moça. All maps are shown in UTM projection (zone 23S, WGS84 datum).

Currently, the species presents high climatic suitability across several areas of the IQ. However, this suitability consistently decreases under future climate scenarios, from the optimistic SSP126 to the pessimistic SSP585, and from the 2050 to the 2090 projections (Figure 3). Consequently, the potential distribution area is largest under current conditions (6,606.79 km^2^), followed by the optimistic scenario in 2050 (Supplementary Table 6). Alarmingly, under the pessimistic scenario in 2090, the species is no longer predicted to persist in the IQ (Figures 3 and S1). Considering the last scenario in which the species still occurs (SSP585 in 2050), several mountain ranges and synclines emerge as priority areas for preservation and restoration, including the Sinclinal Moeda, Serra do Curral, Serra do Gandarela, Serra do Caraça, and the region between Serra de Ouro Branco and Serra de Ouro Preto (Figure 4).

#### 3.2.2 Dyckia consimilis

Unlike other species modeled, *D. consimilis* persists across all scenarios (Figure 3). Its current distribution covers 5,712.18 km^2^ and expands to 6090.42 km^2^ under SSP126 in 2050, before declining thereafter (Table S6). Even under the pessimistic SSP585 in 2090, the species maintains 338.09 km^2^ of suitable area within the IQ (Figure S1). Key refugia for conservation include the Sinclinal Moeda and Serra do Caraça, which remain suitable across both optimistic and pessimistic futures (Figure 4).

#### 3.2.3 Dyckia rariflora

In the IQ, 3,185.75 km^2^ are projected as suitable under present conditions (Figure 3 and Table S6). A sharp reduction is projected under future scenarios, with suitable areas declining to 435.26 km^2^ under SSP126 and 652.09 km^2^ under SSP585 in 2050. By 2090, the species is projected to retain only 704.29 km^2^ under SSP126, while it disappears completely under SSP585 (Figure S1). Despite this contraction, transient expansions occur in 2050, with distribution shifting from the southern to the northern portion of the eastern IQ. Considering the distributions under SSP585 in 2050 and SSP126 in 2090, the Serra do Caraça and the southern region of Serra de Ouro Preto stand out as strategic areas for conservation and restoration actions with the species (Figure 4).

#### 3.2.4 Hoplocryptanthus ferrarius

The present distribution of the species covers 4,867.36 km^2^ in the IQ (Table S6). Under the optimistic scenario (SSP126) the distribution contracts strongly by 2050 (1,227.88 km^2^) but shows a slight expansion again in 2090 (1,431.86 km^2^), with the same distribution shifting as *D. rariflora*, from the southern to the northern portion of the eastern IQ. Under the pessimistic scenario (SSP585; Figure 3), the distribution shrinks to 606.31 km^2^ in 2050 and disappears completely by 2090 due to the threshold cut‐off (Figure S1). Considering these projections, priority areas for conservation include the Sinclinal Moeda, Serra do Curral, and Serra de Ouro Branco (Figure 4).

#### 3.2.5 Hoplocryptanthus schwackeanus

According to the distribution maps, climatic suitability for *H. schwackeanus* decreases progressively across scenarios and decades. Nevertheless, suitable areas above 0.5 remain even under the pessimistic scenario (SSP585) in 2090 (Figure 3). Currently, the species occupies 7,856.36 km^2^ in the IQ, with the second largest distribution projected under SSP126 in 2050 (Table S6). Under SSP585 in 2090, however, its distribution is reduced to the lowest values, representing a severe contraction (Figure S1). Under this most critical scenario, the Serra do Caraça emerges as a priority area for conservation and potential reintroduction (Figure 4).

#### 3.2.6 Paepalanthus amoenus

The distribution area decreases markedly under both scenarios by 2050, with a slightly larger extent under the pessimistic scenario (SSP585; 5,162.89 km^2^) than under the optimistic one (SSP126; 4,982.20 km^2^; Table S6). By 2090, however, the trends diverge: under SSP126 the species recovers part of its distribution (5,675.24 km^2^; Figure 3), while under SSP585 it undergoes a sharp reduction, persisting only in small remnants (92.35 km^2^; Figure S1). Under this most critical scenario, priority areas for conservation and potential reintroduction are located from Serra de Ouro Branco to Serra do Gandarela (Figure 4).

#### 3.2.7 Paepalanthus magalhaesii

The species showed a progressive reduction in climatic suitability across scenarios and decades, although suitable areas remain present under different conditions (Figure 3). Its distribution decreased from 3,161.65 km^2^ under current conditions to only 3.21 km^2^ under the pessimistic scenario (SSP585) in 2090 (Table S6). Until 2050, including under SSP585, the species persists across several mountain ranges of the IQ, but becomes highly restricted by 2090 (Figure S1). Under this most critical scenario, the Serra do Caraça emerges as a key area for conservation and restoration (Figure 4).

#### 3.2.8 Vriesea minarum

Climatic suitability decreases under both scenarios. However, under the optimistic scenario (SSP126), the distribution contracts slightly in 2050 (6,184.38 km^2^) but expands again in 2090, surpassing the current extent (6,523.27 km^2^; Table S6 and Figure 3). In contrast, under the pessimistic scenario (SSP585), the distribution shrinks considerably in 2050 (4,220.89 km^2^) and declines sharply to only 718.74 km^2^ by 2090 (Figure S1). Considering the last scenario in which the species still persists (SSP585 in 2090), several mountain ranges and synclines of the IQ emerge as priority areas for conservation and restoration: the southern Sinclinal Moeda, Serra do Gandarela, Serra do Caraça, the region between Serra de Ouro Branco and Serra de Ouro Preto, and a small area in Serra da Piedade (Figure 4).

### 3.3 Comparative Response Patterns Across Species

When comparing species responses across scenarios, range contractions under SSP585 were the dominant pattern by 2090. Three species ( *A. tetragona* , *D. rariflora*, and *H. ferrarius*) were projected to lose all climatically suitable area within the IQ, while *P. magalhaesii* was reduced to 3.21 km^2^. In contrast, *D. consimilis* and *H. schwackeanus* retained measurable suitable areas across all scenarios, although substantially reduced relative to present conditions (Figure 4).

Species differed in the timing and magnitude of decline. Some taxa showed pronounced contractions by 2050 (e.g., *H. ferrarius*), whereas others maintained broader extents under SSP126 but declined under SSP585 (e.g., *V. minarum* and *P. amoenus* ). Initial distribution size was not consistently associated with persistence under future scenarios: for example, *A. tetragona* , currently occupying more than 6,600 km^2^, was projected to disappear, whereas *D. consimilis* retained residual suitability despite a smaller initial extent.

Several species exhibited temporary redistribution toward the northern and eastern sectors of the IQ under intermediate scenarios; however, these shifts were not maintained under the most restrictive projections.

### 3.4 Identification of Priority Areas for Conservation

**FIGURE 5:**
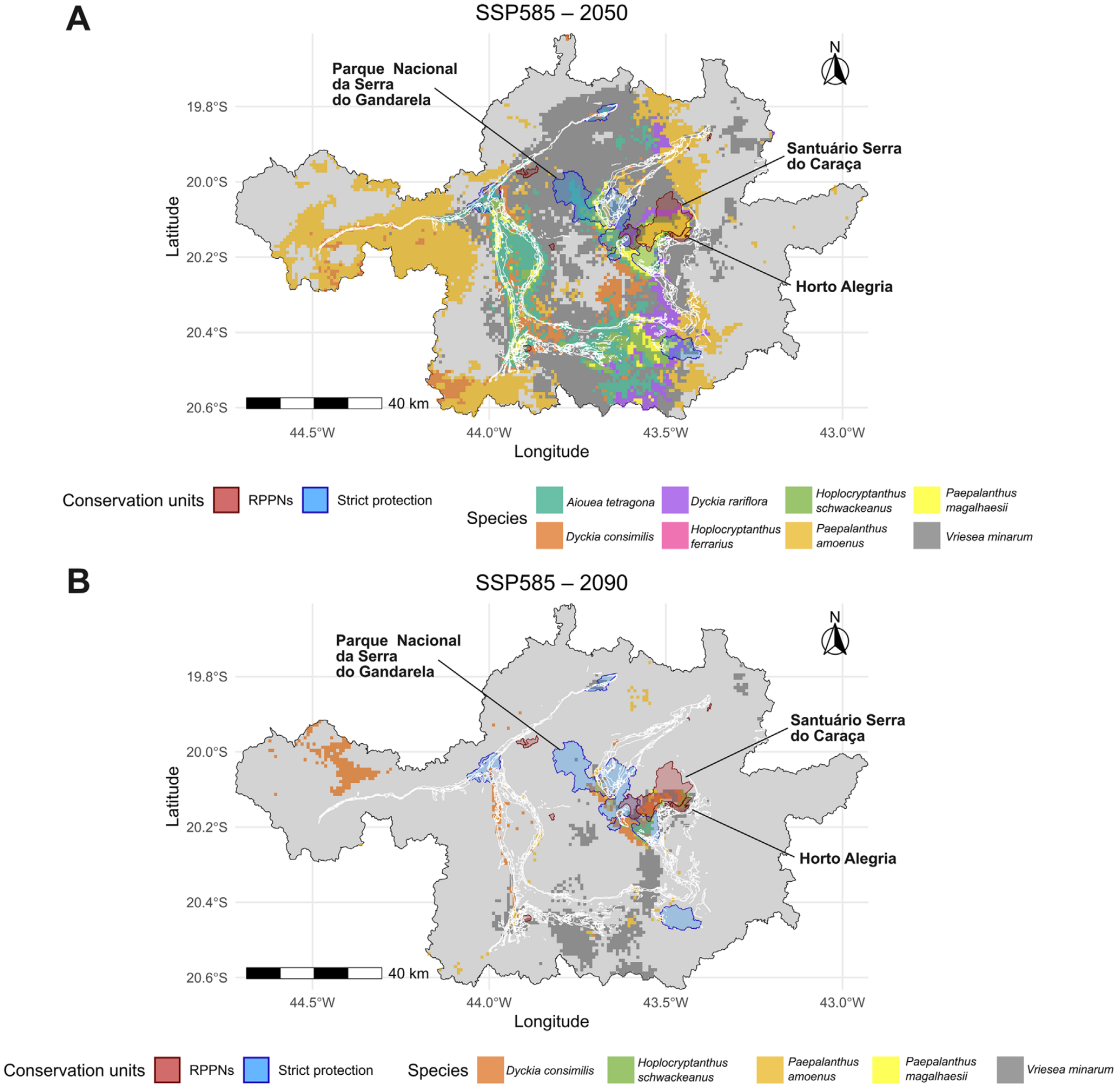
Critical maps showing the spatial distribution of the eight studied species under the most restrictive occurrence scenario (SSP585) for: (A) 2050 (2041–2060) and (B) 2090 (2081–2100). Colored areas indicate species presence, allowing visualization of spatial overlap. Dark red polygons represent private natural heritage reserves (RPPNs), while blue polygons represent strict protection areas. White lines depict the geology of the IQ. Maps are displayed in UTM projection (zone 23S, WGS84 datum).

Considering SSP585 projections separately for mid‐century (2050) and end‐century (2090), we generated two conservation‐overlap maps showing the potential distribution of all modeled species relative to protected areas (Figure 5A,B). In 2050, all eight species overlap with strict protection units or RPPNs, particularly within Parque Nacional Serra do Gandarela, which concentrates the largest continuous area of suitable habitat. Overlap is also observed in the RPPN Santuário Serra do Caraça and other protected areas, forming a connected corridor of suitable habitats extending from Serra de Ouro Branco through Gandarela to Caraça. Important suitable areas are also identified in the Sinclinal Moeda region. By 2090, suitable habitats become more spatially concentrated. Although Gandarela and Caraça remain the primary refuges, suitable areas are still detected in Horto Alegria and parts of the Sinclinal Moeda. However, the spatial continuity observed in 2050 appears reduced, indicating a progressive contraction and fragmentation of climatically suitable environments under an intensified scenario.

**FIGURE 6:**
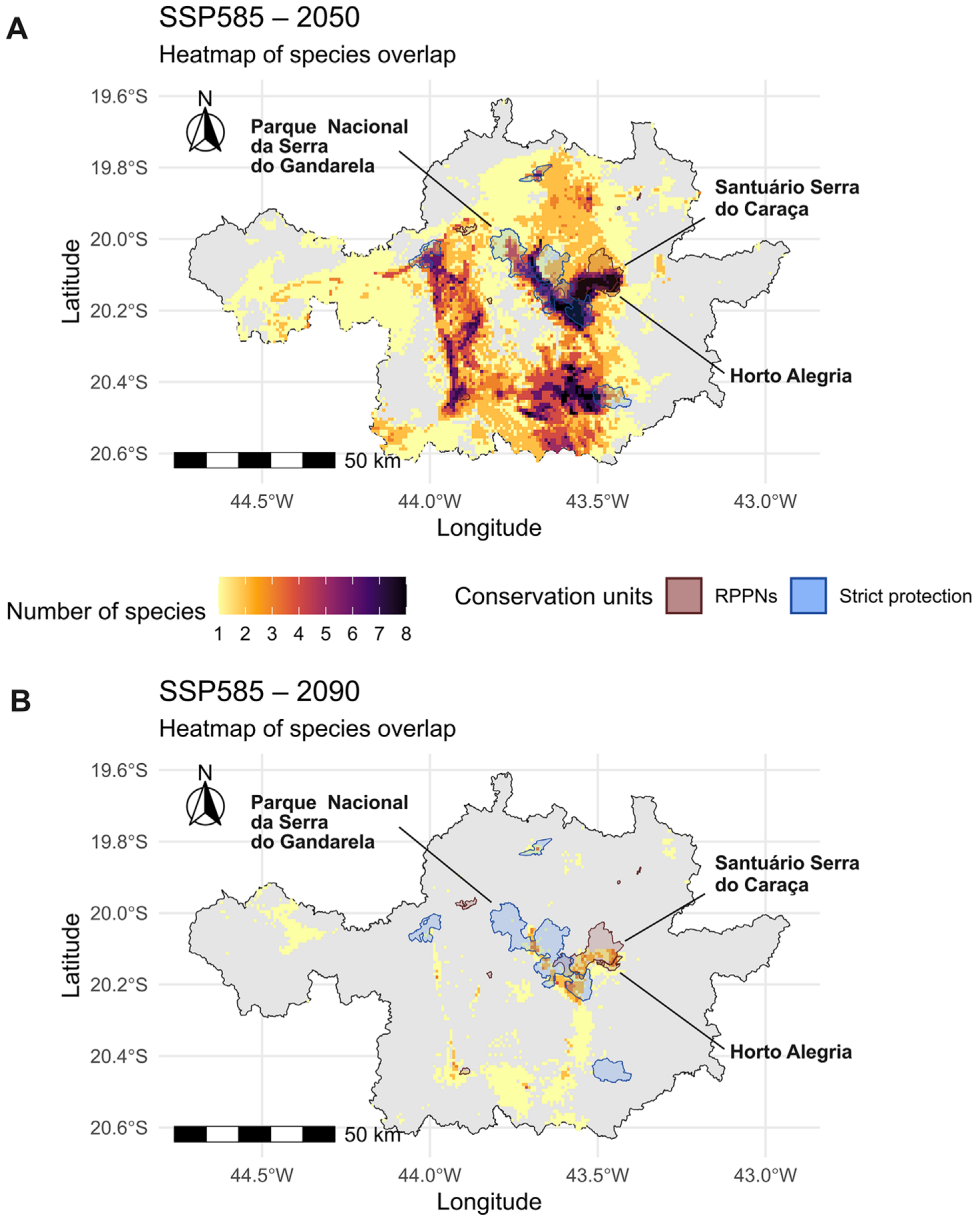
Heatmaps representing the spatial overlap of the eight species under the SSP585 climate scenario for (A) 2050 (2041–2060) and (B) 2090 (2081–2100). The color gradient indicates the number of species present per pixel (from 1 to 8). Black lines delimit the study area, dark red polygons represent private natural heritage reserves (RPPNs), and blue polygons represent strict protection areas. Maps are displayed in UTM projection (zone 23S, WGS84 datum).

To complement this spatial assessment, we produced heatmaps of species overlap (Figure 6A,B), emphasizing co‐occurrence intensity rather than individual distributions. Darker colors represent a higher number of overlapping species. In 2050, the Gandarela‐Caraça axis clearly emerges as the dominant hotspot, with up to eight species predicted to co‐occur. Additional high‐overlap areas (> 7 species) are detected in Serra de Ouro Branco and portions of the Sinclinal Moeda, although these are only partially covered by strict protection units. By 2090, overlap intensity becomes more restricted. The Gandarela‐Caraça axis maintains co‐occurrence of at least four species, reinforcing its importance as a climatic refuge. Hotspots with four overlapping species are also observed in the RPPN Horto Alegria. Additional fragmented areas supporting three of the five persisting species are identified in Serra do Ouro Preto and in the southern Sinclinal Moeda suggesting potential targets for future expansion of strict protection units.

## 4 Discussion

Considering that the ensemble models for all species achieved acceptable to excellent predictive performance, we were able to robustly describe their current distributions under present climatic conditions and project their potential future shifts. Our projections indicate a substantial contraction of suitable habitats in the IQ under pessimistic climate scenarios. This pattern indicates high projected sensitivity of rupestrian grassland taxa to future macroclimatic change. Xeromorphic species such as *A. tetragona* , *D*. *rariflora*, *H. ferrarius*, and *P. magalhaesii* are particularly threatened, with models indicating their disappearance from the region under SSP585 by 2090. In contrast, a limited number of species demonstrated relative resilience under the most pessimistic scenarios, including *D. consimilis* and *H. schwackeanus*, while others even exhibited a transient expansion of suitable habitat in at least one future projection, including *P. amoenus* and *V. minarum*. Still, habitat loss was a prevailing outcome. For instance, *D. rariflora* is projected to lose over 86% of its current distribution by 2050, whereas *P. magalhaesii* suffers a near‐complete collapse, retaining only a few square kilometers by 2090 under SSP585.

Similar patterns of dramatic reductions in suitable habitats under global warming have been reported for other xeromorphic species. For instance, the endemic and vulnerable Ethiopian tree *Vachellia negrii* is projected to lose up to 758,325 ha of habitat, while the endangered savanna tree *Pterocarpus erinaceus* in Burkina Faso may lose up to 61% of its highly suitable areas by 2070 (Dimobe et al. 2022; Semu et al. 2021). Likewise, Brazilian rupicolous bromeliads such as *Dyckia dissitiflora* and *D. pernambucana* are projected to undergo regional extinction by 2080, following the complete disappearance of suitable habitats in the Chapada Diamantina, northeastern Brazil (Fagundes et al. 2025).

These convergent patterns across distant ecosystems suggest that range contractions are not merely stochastic outcomes of global warming but are also influenced by the biological and ecological characteristics of the studied taxa. Differences in reproductive strategies, dispersal potential, population genetic structure, and physiological tolerance during critical stages (e.g., germination and flowering) have been proposed as key determinants of whether species are able to persist under future climates or are driven to regional extinction (Loreto and Atzori 2024; Renton et al. 2012).

Trees and shrubs such as *A. tetragona* , whose broad climatic suitability across the IQ collapses to extinction under the pessimistic scenario in 2090, may face additional constraints related to life‐history traits such as longer generation times and dependence on animal‐mediated pollination, potentially limiting their capacity to track rapidly shifting climatic conditions (Garavito et al. 2015).

Extreme edaphic specialization, fundamental for the persistence of rupestrian flora in rare, nutrient‐poor substrates, confines many bromeliads, including *Dyckia*, *Hoplocryptanthus*, and *Vriesea*, to ferruginous or quartzitic outcrops, thereby severely constraining their ability to follow shifting climates. Although several of these species, along with representatives of *Paepalanthus*, produce wind‐dispersed seeds, their strict substrate dependence sharply limits the extent of climatically suitable habitats where successful establishment is possible (Corlett and Tomlinson 2020). This interaction between narrow edaphic affinity and restricted colonization potential underscores a critical paradox: even taxa equipped with efficient dispersal mechanisms and notable drought resistance may remain disproportionately vulnerable to regional extinction under future climate scenarios (Cramer et al. 2022; Rajakaruna 2018).

Beyond their extreme edaphic dependence, bromeliads are additionally constrained by physiological limitations inherent to their carbon‐concentrating metabolism. Although CAM and C4 pathways are typically regarded as hallmarks of drought adaptation, their efficiency declines under warming conditions, particularly with rising nocturnal temperatures (Leverett and Borland 2023). Night‐time warming elevates respiration and evapotranspiration during the CO_2_ fixation phase, thereby reducing the efficiency of CAM metabolism, intensifying physiological stress, and ultimately impairing key processes such as flowering and responses to environmental stress (Winter and Smith 2022; Yang et al. 2015). While our models do not explicitly incorporate microclimatic buffering or physiological tolerance, previously documented traits such as edaphic specialization and carbon‐concentrating metabolism may help contextualize the projected distributional patterns (Figure 4).

In the long‐term, the persistence of rupestrian species will largely depend on the effectiveness of conservation strategies within the IQ. Despite the region's abundance of conservation units, the diversity, genetic structure, and ecological dynamics of many endemic taxa remain poorly documented, with numerous species still undescribed, thereby constraining the development of targeted management actions (Lamounier et al. 2011; Lobo and Cioni 2024).

Among these units, the Parque Nacional da Serra do Gandarela stands out as the largest reserve in the region, encompassing extensive rupestrian landscapes, including ferruginous outcrops, and, according to our projections, retaining future suitable habitats for several species even under the most pessimistic scenarios (e.g., *D. consimilis* , *H. schwackeanus*, *P. amoenus* , and *V. minarum*; Figure 5B). Although several protected areas overlap with projected refugia, their long‐term effectiveness will depend on management capacity and surrounding land‐use dynamics (Ministério do Meio Ambiente (Brasil), Instituto Chico Mendes de Conservação da Biodiversidade (ICMBio) 2024).

While current management challenges may undermine the effectiveness of protected areas such as Parque Nacional da Serra do Gandarela, their strategic importance is reinforced by their potential role as future climatic refugia. These areas not only ensure the continuity of rupestrian species under warming scenarios but also mirror the historical function of montane strongholds within the Espinhaço Range. During Pleistocene climatic oscillations, “sky islands” across the range acted as safe havens, allowing species to survive adverse periods and later expand and radiate during phases of climatic amelioration (Esser et al. 2024; Magri et al. 2025) identifying present‐day climatic strongholds and assessing their representation within the existing network of conservation units are therefore crucial steps to safeguard rupestrian biodiversity under future climate change.

The climate projections used in this study are based on the CMIP6 framework and the SSPs, which combine socioeconomic narratives (SSP1–SSP5) with different levels of radiative forcing (O'Neill, Kriegler, et al. 2017; IPCC 2021). SSP1‐2.6 (SSP126) represents a sustainability‐oriented pathway with strong mitigation and low greenhouse gas emissions, whereas SSP5‐8.5 (SSP585) reflects a fossil‐fueled development trajectory associated with high emissions. The divergence between these pathways has been consistently demonstrated in CMIP6 global projections, particularly regarding temperature increases and climate extremes by the end of the century. Within this context, our models identify potential areas of persistence under SSP585 in both mid‐century and end‐century projections, concentrated along the Gandarela–Caraça axis. Within this corridor, high‐elevation sites such as the RPPN Santuário Serra do Caraça (~2072 m), RPPN Horto Alegria, and Serra do Gandarela emerge as climatically stable zones (Figure 6A,B), reinforcing their importance as potential refugia under intensified warming.

However, the effectiveness of these refugia is constrained by the strict edaphic requirements of rupestrian taxa. In ferruginous and quartzitic environments, the absence of continuous substrates prevents the formation of uninterrupted altitudinal gradients, restricting opportunities for upward migration. Consequently, only populations already established at higher elevations are more likely to persist, while small and isolated groups face heightened risks of genetic erosion, demographic bottlenecks, and reduced adaptive potential (Kou et al. 2024; Ren et al. 2020).

These projections highlight the potential need for active conservation interventions, particularly in landscapes identified as persistent suitability zones. Our modeling results provide a valuable framework to guide proactive conservation actions by highlighting areas of opportunity for both the discovery of new populations and the reorientation of in situ strategies, which have so far proved challenging (Fernandes et al. 2014; Pilon et al. 2023). The models also support planned translocation initiatives, moving individuals or propagules to areas with higher probabilities of remaining suitable under future climates. Among these, the Gandarela–Caraça corridor consistently emerges as a priority for such initiatives, given its role as an overlap zone for multiple species in our projections. By combining predictive modeling with ecological and genetic information, conservation programs can be expanded to include *ex situ* cultivation and ecosystem restoration. Together, these measures are crucial to anticipate biodiversity loss before natural populations collapse.

Nevertheless, our results reinforce the importance of landscape‐level conservation planning within the IQ. The persistence of rupestrian biodiversity depends not only on isolated climatic refugia but also on maintaining habitat connectivity across altitudinal gradients and heterogeneous substrates. Ensuring the protection of ecological corridors and integrating conservation units into broader territorial planning frameworks are essential to sustain ecosystem processes, genetic connectivity, and long‐term evolutionary potential.

Finally, the interactive application developed in this study provides managers and policymakers with an accessible tool to explore distributions, assess risks, and identify priority areas for action, turning complex modeling outputs into practical guidance for decision‐making. Together, these results emphasize that climate mitigation alone will not suffice; integrated and forward‐looking conservation strategies will be essential to safeguard of rupestrian biodiversity in the IQ.

## 5 Conclusions

Our results indicate that rupestrian species of the Iron Quadrangle may be highly sensitive to projected macroclimatic changes, as reflected by substantial reductions in climatically suitable areas under pessimistic scenarios.

By modeling current distributions and projecting future shifts, we identified potential climatic strongholds. In particular, the Gandarela‐Caraça corridor stood out as a priority landscape for conservation, where target actions (e.g., ecological connectivity and planning translocation) could help mitigate biodiversity loss. Together, these findings provide practical guidance for in situ conservations and emphasize that climate mitigation alone will not suffice; integrated, forward‐looking strategies will be essential to ensure the persistence of rocky plant diversity in the Iron Quadrangle.

## Author Contributions

**Ana Flávia Francisconi:** conceptualization (lead), data curation (lead), formal analysis (lead), investigation (lead), methodology (lead), visualization (lead), writing – original draft (lead), writing – review and editing (lead). **Maria Imaculada Zucchi:** conceptualization (equal), funding acquisition (equal), investigation (equal), project administration (equal), resources (equal), supervision (equal), writing – original draft (equal). **João Victor da Silva Rabelo‐Araujo:** conceptualization (lead), data curation (lead), formal analysis (lead), investigation (lead), methodology (lead), visualization (lead), writing – original draft (lead), writing – review and editing (lead). **Ana Cristina Silva Amoroso Anastacio:** conceptualization (equal), funding acquisition (equal), writing – review and editing (equal). **Ana Paula da Silva Marques:** investigation (equal), writing – review and editing (equal).

## Funding

This work was supported by Agência Paulista de Tecnologia dos Agronegócios (2022.2658), Fundação de Amparo à Pesquisa do Estado de São Paulo (10.13039/501100001807) (2023/00765‐9).

## Conflicts of Interest

This study was conducted in partnership with Vale S.A., which provided occurrence datasets and contributed co‐authors employed by the company. Although Vale S.A. is a mining company with active operations in the Iron Quadrangle, all analyses, interpretations, and conclusions presented here were conducted independently by the research team and are not influenced by the company's commercial activities. The authors declare no other conflicts of interest.

## Supporting information


**Table S1:** Species included in the ecological niche models and their corresponding synonyms, according to Reflora (https://floradobrasil.jbrj.gov.br/reflora/PrincipalUC/PrincipalUC.do?lingua=pt).
**Table S2:** Occurrence records of the studied species, compiled from online herbaria and research datasets retained after filtering, and used in ecological niche modeling (ENM), after rarefaction.
**Table S3:** Occurrence records of the studied species, compiled from online herbaria and research datasets retained after filtering.
**Table S4:** Explained variance of principal components derived from the 19 WorldClim bioclimatic variables used in ENMTML.
**Table S5:** Loadings of the 19 WorldClim bioclimatic variables (Bio1–Bio19) on the first four principal components (PC1–PC4) retained for ecological niche modeling in ENMTML. Values represent the correlation coefficients between original variables and each principal component. The first four components cumulatively explained 96.8% of the total variance.
**Table S6:** Projected suitable distribution areas (km^2^) for each species in the Iron Quadrangle under current conditions and future climate scenarios (SSP126 and SSP585 for 2050 and 2090).
**Figure S1:** Current and projected distributions of eight plant species in the Iron Quadrangle (IQ) under climate change scenarios. Each row represents one species (*Aiouea tetragona, Dyckia consimilis, Dyckia rariflora*, *Hoplocryptanthus ferrarius, Hoplocryptanthus schwackeanus*, *Paepalanthus amoenus*, *Paepalanthus magalhaesii*, and *Vriesea minarum*). Columns show the current distribution (dark gray) overlaid with projections for SSP126 2050 (light green), SSP126 2090 (dark green), SSP585 2050 (light purple), and SSP585 2090 (dark purple). Suitable areas are displayed in binary maps (presence = 1; absence = 0).

## Data Availability

The data that supports the findings of this study are available in the Supporting Information of this article. Occurrence records used in this study were obtained from publicly available online herbaria (e.g., speciesLink, GBIF) and from research partnership agreements, and are available in Table S3. Climate data layers (bio1–bio19) were downloaded from WorldClim v2.1 (https://www.worldclim.org). Future climate projections were obtained from CMIP6 global circulation models (EC‐EARTH3‐veg, INM‐CM5‐0, and MRI‐ESM2‐0). An interactive Shiny application is also available at https://labgen.shinyapps.io/ENMBrIQ/, allowing users to visualize current and future distributions, critical scenarios, and conservation priority areas.
