# Supplementary material for: Species Distribution Modeling Reveals Future Climate Refugia and Important Areas for Rocky Plants in Brazil's Iron Quadrangle
Source: Ecol Evol. 2026 Apr 5;16(4):e73355. doi: 10.1002/ece3.73355 (PMC13051957; doi:10.1002/ece3.73355)
Supplement: Supplementary file 1 — Table S1: Species included in the ecological niche models and their corresponding synonyms, according to Reflora (https://floradobrasil.jbrj.gov.br/reflora/PrincipalUC/PrincipalUC.do?lingua=pt). Table S2: Occurrence records of the studied species, compiled from online herbaria and research datasets retained after filtering, and used in ecological niche modeling (ENM), after rarefaction. Table S3: Occurrence records of the studied species, compiled from online herbaria and research datasets retained after filtering. Table S4: Explained variance of principal components derived from the 19 WorldClim bioclimatic variables used in ENMTML. Table S5: Loadings of the 19 WorldClim bioclimatic variables (Bio1–Bio19) on the first four principal components (PC1–PC4) retained for ecological niche modeling in ENMTML. Values represent the correlation coefficients between original variables and each principal component. The first four components cumulatively explained 96.8% of the total variance. Table S6: Projected suitable distribution areas (km2) for each species in the Iron Quadrangle under current conditions and future climate scenarios (SSP126 and SSP585 for 2050 and 2090). Figure S1: Current and projected distributions of eight plant species in the Iron Quadrangle (IQ) under climate change scenarios. Each row represents one species (Aiouea tetragona, Dyckia consimilis, Dyckia rariflora, Hoplocryptanthus ferrarius, Hoplocryptanthus schwackeanus, Paepalanthus amoenus, Paepalanthus magalhaesii, and Vriesea minarum). Columns show the current distribution (dark gray) overlaid with projections for SSP126 2050 (light green), SSP126 2090 (dark green), SSP585 2050 (light purple), and SSP585 2090 (dark purple). Suitable areas are displayed in binary maps (presence = 1; absence = 0). [file ECE3-16-e73355-s001.docx]

**Supplementary Material: Species distribution modeling reveals future climate refugia and important areas for rocky plants in Brazil's Iron Quadrangle**

Supplementary Table 1. Species included in the ecological niche models and their corresponding synonyms, according to Reflora (<https://floradobrasil.jbrj.gov.br/reflora/PrincipalUC/PrincipalUC.do?lingua=pt>)

| Species | Synonyms |
| --- | --- |
| *Aiouea tetragona* | *Cinnamomum quadrangulum, Oreodaphne tetragona* |
| *Dyckia consimilis* | *Dyckia consimilis Mez* |
| *Dyckia rariflora* | *Dyckia elata* |
| *Hoplocryptanthus ferrarius* | *Cryptanthus ferrarius* |
| *Hoplocryptanthus schwackeanus* | *Cryptanthus schwackeanus* |
| *Paepalanthus amoenus* | *Eriocaulon longipes, Paepalanthus amoenus var. curralensis Silveira, Paepalanthus amoenus var. prolifer Moldenke, Dupatya amoena, Eriocaulon amoenum* |
| *Paepalanthus magalhaesii* | *Paepalanthus gomessi* |
| *Vriesea minarum* | *Vriesea citrina, Vriesea ouroensis* |

Supplementary Table 2 - Occurrence records of the studied species, compiled from online herbaria and research datasets retained after filtering, and used in ecological niche modeling (ENM), after rarefaction.

| Species | Occurrences | Occurrences used in ENM |
| --- | --- | --- |
| *Aiouea tetragona* | 76 | 47 |
| *Dyckia consimilis* | 75 | 39 |
| *Dyckia rariflora* | 41 | 25 |
| *Hoplocryptanthus ferrarius* | 34 | 18 |
| *Hoplocryptanthus schwackeanus* | 131 | 55 |
| *Paepalanthus amoenus* | 65 | 37 |
| *Paepalanthus magalhaesii* | 34 | 12 |
| *Vriesea minarum* | 83 | 40 |

Supplementary Table 3. Occurrence records of the studied species, compiled from online herbaria and research datasets retained after filtering.

| species | longitude | latitude |
| --- | --- | --- |
| *Aiouea tetragona* | -44.675833 | -20.091944 |
| *Aiouea tetragona* | -44.2325 | -20.115556 |
| *Aiouea tetragona* | -44.199722 | -20.143333 |
| *Aiouea tetragona* | -44.199699 | -20.143299 |
| *Aiouea tetragona* | -44.199699 | -20.143299 |
| *Aiouea tetragona* | -44.199699 | -20.143299 |
| *Aiouea tetragona* | -44.15 | -20.100556 |
| *Aiouea tetragona* | -44.052799 | -20.333099 |
| *Aiouea tetragona* | -44.033333 | -20.066667 |
| *Aiouea tetragona* | -44.033333 | -20.044722 |
| *Aiouea tetragona* | -44.028056 | -20.080278 |
| *Aiouea tetragona* | -44.028056 | -20.030278 |
| *Aiouea tetragona* | -44.015033 | -20.057358 |
| *Aiouea tetragona* | -44.002906 | -20.050304 |
| *Aiouea tetragona* | -43.9993 | -20.0531 |
| *Aiouea tetragona* | -43.991389 | -20.077222 |
| *Aiouea tetragona* | -43.983906 | -20.107833 |
| *Aiouea tetragona* | -43.98373 | -20.093041 |
| *Aiouea tetragona* | -43.983611 | -20.093056 |
| *Aiouea tetragona* | -43.983333 | -20.1 |
| *Aiouea tetragona* | -43.9799 | -20.0133 |
| *Aiouea tetragona* | -43.97921 | -20.110477 |
| *Aiouea tetragona* | -43.970386 | -20.119969 |
| *Aiouea tetragona* | -43.966667 | -20.116667 |
| *Aiouea tetragona* | -43.965477 | -20.258724 |
| *Aiouea tetragona* | -43.965278 | -20.2475 |
| *Aiouea tetragona* | -43.958056 | -20.076944 |
| *Aiouea tetragona* | -43.956111 | -20.095556 |
| *Aiouea tetragona* | -43.955556 | -20.271389 |
| *Aiouea tetragona* | -43.9425 | -20.292778 |
| *Aiouea tetragona* | -43.938083 | -20.348444 |
| *Aiouea tetragona* | -43.937801 | -19.920799 |
| *Aiouea tetragona* | -43.937778 | -19.920833 |
| *Aiouea tetragona* | -43.932583 | -20.301806 |
| *Aiouea tetragona* | -43.926944 | -20.13 |
| *Aiouea tetragona* | -43.920833 | -19.966389 |
| *Aiouea tetragona* | -43.920222 | -20.419889 |
| *Aiouea tetragona* | -43.918889 | -20.441111 |
| *Aiouea tetragona* | -43.905894 | -20.285751 |
| *Aiouea tetragona* | -43.905833 | -20.445833 |
| *Aiouea tetragona* | -43.901944 | -20.110556 |
| *Aiouea tetragona* | -43.891111 | -20.105278 |
| *Aiouea tetragona* | -43.89 | -19.945278 |
| *Aiouea tetragona* | -43.87975 | -20.255944 |
| *Aiouea tetragona* | -43.867222 | -20.240556 |
| *Aiouea tetragona* | -43.863333 | -20.445556 |
| *Aiouea tetragona* | -43.854939 | -20.2554 |
| *Aiouea tetragona* | -43.851667 | -20.444444 |
| *Aiouea tetragona* | -43.848611 | -20.228611 |
| *Aiouea tetragona* | -43.801399 | -20.2533 |
| *Aiouea tetragona* | -43.801389 | -20.253333 |
| *Aiouea tetragona* | -43.789398 | -20.0875 |
| *Aiouea tetragona* | -43.685417 | -19.823139 |
| *Aiouea tetragona* | -43.685278 | -19.823056 |
| *Aiouea tetragona* | -43.674167 | -19.8175 |
| *Aiouea tetragona* | -43.669701 | -19.879999 |
| *Aiouea tetragona* | -43.666667 | -19.816667 |
| *Aiouea tetragona* | -43.508099 | -20.2875 |
| *Aiouea tetragona* | -43.498611 | -20.150556 |
| *Aiouea tetragona* | -43.498583 | -20.150611 |
| *Aiouea tetragona* | -43.487202 | -19.945801 |
| *Aiouea tetragona* | -43.484 | -20.154917 |
| *Aiouea tetragona* | -43.469444 | -20.230278 |
| *Aiouea tetragona* | -43.453056 | -20.161667 |
| *Aiouea tetragona* | -43.448333 | -19.882222 |
| *Aiouea tetragona* | -43.437778 | -19.889722 |
| *Aiouea tetragona* | -43.436361 | -19.885556 |
| *Aiouea tetragona* | -43.435556 | -20.197222 |
| *Aiouea tetragona* | -43.432222 | -20.197222 |
| *Aiouea tetragona* | -43.429722 | -20.159722 |
| *Aiouea tetragona* | -43.429101 | -20.142024 |
| *Aiouea tetragona* | -43.428056 | -20.159444 |
| *Aiouea tetragona* | -43.4175 | -20.135 |
| *Aiouea tetragona* | -43.4161 | -20.3778 |
| *Aiouea tetragona* | -43.415556 | -20.159167 |
| *Aiouea tetragona* | -43.407501 | -20.074699 |
| *Aiouea tetragona* | -43.405278 | -20.137361 |
| *Dyckia consimilis* | -44.199699 | -20.143299 |
| *Dyckia consimilis* | -44.052799 | -20.333099 |
| *Dyckia consimilis* | -44.033333 | -20.044722 |
| *Dyckia consimilis* | -44.028056 | -20.080278 |
| *Dyckia consimilis* | -44.027307 | -20.059501 |
| *Dyckia consimilis* | -44.013002 | -20.059759 |
| *Dyckia consimilis* | -44.001111 | -20.077222 |
| *Dyckia consimilis* | -44 | -20.114928 |
| *Dyckia consimilis* | -43.997222 | -20.037222 |
| *Dyckia consimilis* | -43.990833 | -20.100278 |
| *Dyckia consimilis* | -43.985833 | -20.075833 |
| *Dyckia consimilis* | -43.982236 | -20.115699 |
| *Dyckia consimilis* | -43.976945 | -20.010833 |
| *Dyckia consimilis* | -43.9725 | -20.136131 |
| *Dyckia consimilis* | -43.971464 | -20.120303 |
| *Dyckia consimilis* | -43.97 | -20.3 |
| *Dyckia consimilis* | -43.966111 | -20.258333 |
| *Dyckia consimilis* | -43.966105 | -20.258339 |
| *Dyckia consimilis* | -43.965278 | -20.2475 |
| *Dyckia consimilis* | -43.963815 | -20.263513 |
| *Dyckia consimilis* | -43.962794 | -20.273354 |
| *Dyckia consimilis* | -43.960556 | -20.09 |
| *Dyckia consimilis* | -43.956389 | -20.273056 |
| *Dyckia consimilis* | -43.950556 | -20.293694 |
| *Dyckia consimilis* | -43.95 | -20.294444 |
| *Dyckia consimilis* | -43.938083 | -20.348444 |
| *Dyckia consimilis* | -43.937801 | -19.920799 |
| *Dyckia consimilis* | -43.937503 | -20.332236 |
| *Dyckia consimilis* | -43.930278 | -20.421389 |
| *Dyckia consimilis* | -43.915278 | -20.154444 |
| *Dyckia consimilis* | -43.9075 | -19.958056 |
| *Dyckia consimilis* | -43.893611 | -19.946944 |
| *Dyckia consimilis* | -43.891111 | -20.105278 |
| *Dyckia consimilis* | -43.888611 | -20.281389 |
| *Dyckia consimilis* | -43.888333 | -20.280833 |
| *Dyckia consimilis* | -43.888319 | -20.280888 |
| *Dyckia consimilis* | -43.887939 | -20.281133 |
| *Dyckia consimilis* | -43.885833 | -19.948056 |
| *Dyckia consimilis* | -43.885278 | -20.278056 |
| *Dyckia consimilis* | -43.885 | -20.2775 |
| *Dyckia consimilis* | -43.883543 | -20.274199 |
| *Dyckia consimilis* | -43.883347 | -20.27366 |
| *Dyckia consimilis* | -43.88189 | -20.27023 |
| *Dyckia consimilis* | -43.879388 | -20.26651 |
| *Dyckia consimilis* | -43.858889 | -20.447222 |
| *Dyckia consimilis* | -43.85887 | -20.447088 |
| *Dyckia consimilis* | -43.848935 | -20.228917 |
| *Dyckia consimilis* | -43.848889 | -20.23 |
| *Dyckia consimilis* | -43.846699 | -19.9856 |
| *Dyckia consimilis* | -43.801399 | -20.2533 |
| *Dyckia consimilis* | -43.693056 | -20.274167 |
| *Dyckia consimilis* | -43.687222 | -20.150833 |
| *Dyckia consimilis* | -43.678611 | -20.229722 |
| *Dyckia consimilis* | -43.6775 | -20.23 |
| *Dyckia consimilis* | -43.676389 | -20.228889 |
| *Dyckia consimilis* | -43.675 | -20.163056 |
| *Dyckia consimilis* | -43.673611 | -20.165 |
| *Dyckia consimilis* | -43.671389 | -20.1675 |
| *Dyckia consimilis* | -43.669722 | -20.218333 |
| *Dyckia consimilis* | -43.668889 | -20.205833 |
| *Dyckia consimilis* | -43.668056 | -20.223889 |
| *Dyckia consimilis* | -43.667778 | -20.223889 |
| *Dyckia consimilis* | -43.665556 | -20.156111 |
| *Dyckia consimilis* | -43.659167 | -20.150556 |
| *Dyckia consimilis* | -43.658056 | -20.152778 |
| *Dyckia consimilis* | -43.631111 | -20.147778 |
| *Dyckia consimilis* | -43.5975 | -20.201111 |
| *Dyckia consimilis* | -43.594167 | -20.21 |
| *Dyckia consimilis* | -43.593922 | -20.209526 |
| *Dyckia consimilis* | -43.583889 | -20.218333 |
| *Dyckia consimilis* | -43.583634 | -20.217909 |
| *Dyckia consimilis* | -43.583611 | -20.219445 |
| *Dyckia consimilis* | -43.583056 | -20.219722 |
| *Dyckia consimilis* | -43.582874 | -20.219407 |
| *Dyckia consimilis* | -43.415298 | -19.9594 |
| *Dyckia rariflora* | -43.355833 | -19.851667 |
| *Dyckia rariflora* | -43.368361 | -19.853333 |
| *Dyckia rariflora* | -43.405278 | -20.137361 |
| *Dyckia rariflora* | -43.406389 | -20.165833 |
| *Dyckia rariflora* | -43.407501 | -20.074699 |
| *Dyckia rariflora* | -43.411389 | -20.226111 |
| *Dyckia rariflora* | -43.414444 | -20.3525 |
| *Dyckia rariflora* | -43.415556 | -20.159167 |
| *Dyckia rariflora* | -43.4161 | -20.3778 |
| *Dyckia rariflora* | -43.419167 | -20.313889 |
| *Dyckia rariflora* | -43.419722 | -20.133056 |
| *Dyckia rariflora* | -43.426111 | -20.1575 |
| *Dyckia rariflora* | -43.428319 | -20.141903 |
| *Dyckia rariflora* | -43.429508 | -20.14784 |
| *Dyckia rariflora* | -43.430245 | -20.148294 |
| *Dyckia rariflora* | -43.430278 | -20.148333 |
| *Dyckia rariflora* | -43.430556 | -20.146944 |
| *Dyckia rariflora* | -43.431488 | -20.145882 |
| *Dyckia rariflora* | -43.432222 | -20.197222 |
| *Dyckia rariflora* | -43.433333 | -20.198611 |
| *Dyckia rariflora* | -43.436667 | -20.153611 |
| *Dyckia rariflora* | -43.4375 | -20.163889 |
| *Dyckia rariflora* | -43.448333 | -19.882222 |
| *Dyckia rariflora* | -43.453056 | -20.161667 |
| *Dyckia rariflora* | -43.479167 | -20.342222 |
| *Dyckia rariflora* | -43.487202 | -19.945801 |
| *Dyckia rariflora* | -43.5018 | -20.354617 |
| *Dyckia rariflora* | -43.503333 | -20.358889 |
| *Dyckia rariflora* | -43.5054 | -20.383861 |
| *Dyckia rariflora* | -43.508099 | -20.2875 |
| *Dyckia rariflora* | -43.514722 | -20.154722 |
| *Dyckia rariflora* | -43.515278 | -20.213081 |
| *Dyckia rariflora* | -43.529836 | -20.241268 |
| *Dyckia rariflora* | -43.52998 | -20.241095 |
| *Dyckia rariflora* | -43.534995 | -20.243138 |
| *Dyckia rariflora* | -43.5775 | -20.018333 |
| *Dyckia rariflora* | -43.605 | -20.197222 |
| *Dyckia rariflora* | -43.683111 | -20.093806 |
| *Dyckia rariflora* | -43.683772 | -20.082562 |
| *Dyckia rariflora* | -43.697222 | -20.488333 |
| *Dyckia rariflora* | -43.849167 | -20.230278 |
| *Hoplocryptanthus ferrarius* | -43.483333 | -20.083333 |
| *Hoplocryptanthus ferrarius* | -43.683111 | -20.093806 |
| *Hoplocryptanthus ferrarius* | -43.4025 | -20.13 |
| *Hoplocryptanthus ferrarius* | -43.453056 | -20.161667 |
| *Hoplocryptanthus ferrarius* | -43.960556 | -20.09 |
| *Hoplocryptanthus ferrarius* | -43.550556 | -20.233056 |
| *Hoplocryptanthus ferrarius* | -43.436667 | -20.153611 |
| *Hoplocryptanthus ferrarius* | -43.895278 | -20.446111 |
| *Hoplocryptanthus ferrarius* | -43.669444 | -20.159444 |
| *Hoplocryptanthus ferrarius* | -43.693889 | -20.274167 |
| *Hoplocryptanthus ferrarius* | -43.431389 | -20.142778 |
| *Hoplocryptanthus ferrarius* | -43.487667 | -20.152389 |
| *Hoplocryptanthus ferrarius* | -43.4161 | -20.3778 |
| *Hoplocryptanthus ferrarius* | -43.508099 | -20.2875 |
| *Hoplocryptanthus ferrarius* | -43.431667 | -20.161111 |
| *Hoplocryptanthus ferrarius* | -43.869445 | -20.258056 |
| *Hoplocryptanthus ferrarius* | -43.419444 | -20.133611 |
| *Hoplocryptanthus ferrarius* | -43.8675 | -20.2675 |
| *Hoplocryptanthus ferrarius* | -43.883889 | -20.275556 |
| *Hoplocryptanthus ferrarius* | -43.589444 | -20.211944 |
| *Hoplocryptanthus ferrarius* | -43.435556 | -20.145833 |
| *Hoplocryptanthus ferrarius* | -43.428889 | -20.141944 |
| *Hoplocryptanthus ferrarius* | -43.495886 | -20.253406 |
| *Hoplocryptanthus ferrarius* | -43.495311 | -20.254425 |
| *Hoplocryptanthus ferrarius* | -43.884534 | -20.274579 |
| *Hoplocryptanthus ferrarius* | -43.883403 | -20.274127 |
| *Hoplocryptanthus ferrarius* | -43.883577 | -20.275202 |
| *Hoplocryptanthus ferrarius* | -43.88163 | -20.269803 |
| *Hoplocryptanthus ferrarius* | -43.880432 | -20.267879 |
| *Hoplocryptanthus ferrarius* | -43.879949 | -20.267162 |
| *Hoplocryptanthus ferrarius* | -43.683371 | -20.090217 |
| *Hoplocryptanthus ferrarius* | -43.434095 | -20.144033 |
| *Hoplocryptanthus ferrarius* | -43.428862 | -20.141988 |
| *Hoplocryptanthus ferrarius* | -43.435641 | -20.145724 |
| *Hoplocryptanthus schwackeanus* | -43.406111 | -20.163611 |
| *Hoplocryptanthus schwackeanus* | -43.406389 | -20.165833 |
| *Hoplocryptanthus schwackeanus* | -43.407501 | -20.074699 |
| *Hoplocryptanthus schwackeanus* | -43.4161 | -20.3778 |
| *Hoplocryptanthus schwackeanus* | -43.418056 | -20.37495 |
| *Hoplocryptanthus schwackeanus* | -43.419167 | -20.313889 |
| *Hoplocryptanthus schwackeanus* | -43.419444 | -20.133611 |
| *Hoplocryptanthus schwackeanus* | -43.424167 | -20.34 |
| *Hoplocryptanthus schwackeanus* | -43.431667 | -20.161111 |
| *Hoplocryptanthus schwackeanus* | -43.435556 | -20.198611 |
| *Hoplocryptanthus schwackeanus* | -43.436667 | -20.153611 |
| *Hoplocryptanthus schwackeanus* | -43.474167 | -20.428056 |
| *Hoplocryptanthus schwackeanus* | -43.483333 | -20.083333 |
| *Hoplocryptanthus schwackeanus* | -43.491667 | -20.429167 |
| *Hoplocryptanthus schwackeanus* | -43.4925 | -20.433056 |
| *Hoplocryptanthus schwackeanus* | -43.492778 | -20.1263 |
| *Hoplocryptanthus schwackeanus* | -43.492778 | -20.126389 |
| *Hoplocryptanthus schwackeanus* | -43.505886 | -20.386694 |
| *Hoplocryptanthus schwackeanus* | -43.508099 | -20.2875 |
| *Hoplocryptanthus schwackeanus* | -43.5125 | -19.876389 |
| *Hoplocryptanthus schwackeanus* | -43.513245 | -19.877914 |
| *Hoplocryptanthus schwackeanus* | -43.513333 | -19.877778 |
| *Hoplocryptanthus schwackeanus* | -43.517 | -20.36515 |
| *Hoplocryptanthus schwackeanus* | -43.518889 | -20.014167 |
| *Hoplocryptanthus schwackeanus* | -43.520556 | -20.363889 |
| *Hoplocryptanthus schwackeanus* | -43.5225 | -19.867778 |
| *Hoplocryptanthus schwackeanus* | -43.522778 | -19.869167 |
| *Hoplocryptanthus schwackeanus* | -43.550556 | -20.233056 |
| *Hoplocryptanthus schwackeanus* | -43.5775 | -20.018333 |
| *Hoplocryptanthus schwackeanus* | -43.580556 | -20.198889 |
| *Hoplocryptanthus schwackeanus* | -43.584158 | -20.217821 |
| *Hoplocryptanthus schwackeanus* | -43.584444 | -20.218333 |
| *Hoplocryptanthus schwackeanus* | -43.587292 | -20.21027 |
| *Hoplocryptanthus schwackeanus* | -43.5925 | -20.211944 |
| *Hoplocryptanthus schwackeanus* | -43.592531 | -20.211956 |
| *Hoplocryptanthus schwackeanus* | -43.6025 | -20.195 |
| *Hoplocryptanthus schwackeanus* | -43.630556 | -20.147222 |
| *Hoplocryptanthus schwackeanus* | -43.631028 | -20.147793 |
| *Hoplocryptanthus schwackeanus* | -43.64117 | -20.106246 |
| *Hoplocryptanthus schwackeanus* | -43.648508 | -20.155618 |
| *Hoplocryptanthus schwackeanus* | -43.648611 | -20.155556 |
| *Hoplocryptanthus schwackeanus* | -43.65839 | -20.151218 |
| *Hoplocryptanthus schwackeanus* | -43.662222 | -20.162222 |
| *Hoplocryptanthus schwackeanus* | -43.665502 | -20.112086 |
| *Hoplocryptanthus schwackeanus* | -43.666667 | -19.818056 |
| *Hoplocryptanthus schwackeanus* | -43.666667 | -19.816667 |
| *Hoplocryptanthus schwackeanus* | -43.668333 | -20.159444 |
| *Hoplocryptanthus schwackeanus* | -43.669076 | -20.110286 |
| *Hoplocryptanthus schwackeanus* | -43.669167 | -20.110278 |
| *Hoplocryptanthus schwackeanus* | -43.669444 | -20.159444 |
| *Hoplocryptanthus schwackeanus* | -43.66969 | -20.158575 |
| *Hoplocryptanthus schwackeanus* | -43.669701 | -19.879999 |
| *Hoplocryptanthus schwackeanus* | -43.670104 | -20.159375 |
| *Hoplocryptanthus schwackeanus* | -43.670565 | -20.104644 |
| *Hoplocryptanthus schwackeanus* | -43.673611 | -20.165278 |
| *Hoplocryptanthus schwackeanus* | -43.674021 | -19.823026 |
| *Hoplocryptanthus schwackeanus* | -43.675222 | -19.815833 |
| *Hoplocryptanthus schwackeanus* | -43.677778 | -20.038611 |
| *Hoplocryptanthus schwackeanus* | -43.678889 | -19.822222 |
| *Hoplocryptanthus schwackeanus* | -43.683111 | -20.093806 |
| *Hoplocryptanthus schwackeanus* | -43.684722 | -20.062778 |
| *Hoplocryptanthus schwackeanus* | -43.684866 | -20.091015 |
| *Hoplocryptanthus schwackeanus* | -43.685088 | -20.091717 |
| *Hoplocryptanthus schwackeanus* | -43.685202 | -20.089943 |
| *Hoplocryptanthus schwackeanus* | -43.685254 | -19.821487 |
| *Hoplocryptanthus schwackeanus* | -43.691241 | -20.058801 |
| *Hoplocryptanthus schwackeanus* | -43.691278 | -20.056667 |
| *Hoplocryptanthus schwackeanus* | -43.726389 | -19.833056 |
| *Hoplocryptanthus schwackeanus* | -43.741389 | -20.127778 |
| *Hoplocryptanthus schwackeanus* | -43.801399 | -20.2533 |
| *Hoplocryptanthus schwackeanus* | -43.806702 | -19.8864 |
| *Hoplocryptanthus schwackeanus* | -43.846667 | -20.223333 |
| *Hoplocryptanthus schwackeanus* | -43.846699 | -19.9856 |
| *Hoplocryptanthus schwackeanus* | -43.846723 | -20.223212 |
| *Hoplocryptanthus schwackeanus* | -43.848611 | -20.228611 |
| *Hoplocryptanthus schwackeanus* | -43.85 | -20.447778 |
| *Hoplocryptanthus schwackeanus* | -43.878487 | -20.265474 |
| *Hoplocryptanthus schwackeanus* | -43.878611 | -20.265556 |
| *Hoplocryptanthus schwackeanus* | -43.879462 | -20.266785 |
| *Hoplocryptanthus schwackeanus* | -43.879722 | -20.267222 |
| *Hoplocryptanthus schwackeanus* | -43.883601 | -20.125797 |
| *Hoplocryptanthus schwackeanus* | -43.883827 | -20.274408 |
| *Hoplocryptanthus schwackeanus* | -43.884167 | -20.274722 |
| *Hoplocryptanthus schwackeanus* | -43.884345 | -20.275475 |
| *Hoplocryptanthus schwackeanus* | -43.884868 | -20.279613 |
| *Hoplocryptanthus schwackeanus* | -43.885685 | -20.279975 |
| *Hoplocryptanthus schwackeanus* | -43.885764 | -20.447248 |
| *Hoplocryptanthus schwackeanus* | -43.885992 | -20.446907 |
| *Hoplocryptanthus schwackeanus* | -43.886111 | -20.447778 |
| *Hoplocryptanthus schwackeanus* | -43.886389 | -20.447222 |
| *Hoplocryptanthus schwackeanus* | -43.886626 | -20.447079 |
| *Hoplocryptanthus schwackeanus* | -43.886944 | -20.4475 |
| *Hoplocryptanthus schwackeanus* | -43.887164 | -20.280792 |
| *Hoplocryptanthus schwackeanus* | -43.8875 | -20.281111 |
| *Hoplocryptanthus schwackeanus* | -43.888333 | -20.280833 |
| *Hoplocryptanthus schwackeanus* | -43.895151 | -20.446041 |
| *Hoplocryptanthus schwackeanus* | -43.895556 | -20.446389 |
| *Hoplocryptanthus schwackeanus* | -43.895556 | -20.446667 |
| *Hoplocryptanthus schwackeanus* | -43.895653 | -20.446573 |
| *Hoplocryptanthus schwackeanus* | -43.895666 | -20.446662 |
| *Hoplocryptanthus schwackeanus* | -43.895801 | -20.432901 |
| *Hoplocryptanthus schwackeanus* | -43.895833 | -20.432778 |
| *Hoplocryptanthus schwackeanus* | -43.898159 | -20.447901 |
| *Hoplocryptanthus schwackeanus* | -43.898181 | -20.447858 |
| *Hoplocryptanthus schwackeanus* | -43.898611 | -20.448333 |
| *Hoplocryptanthus schwackeanus* | -43.899232 | -20.434183 |
| *Hoplocryptanthus schwackeanus* | -43.899695 | -20.434284 |
| *Hoplocryptanthus schwackeanus* | -43.899886 | -20.434409 |
| *Hoplocryptanthus schwackeanus* | -43.9 | -20.434722 |
| *Hoplocryptanthus schwackeanus* | -43.900278 | -20.434722 |
| *Hoplocryptanthus schwackeanus* | -43.901944 | -20.110556 |
| *Hoplocryptanthus schwackeanus* | -43.903802 | -20.448213 |
| *Hoplocryptanthus schwackeanus* | -43.905833 | -20.445833 |
| *Hoplocryptanthus schwackeanus* | -43.906794 | -19.956949 |
| *Hoplocryptanthus schwackeanus* | -43.910278 | -19.961667 |
| *Hoplocryptanthus schwackeanus* | -43.915098 | -19.962907 |
| *Hoplocryptanthus schwackeanus* | -43.918889 | -20.441111 |
| *Hoplocryptanthus schwackeanus* | -43.937801 | -19.920799 |
| *Hoplocryptanthus schwackeanus* | -43.938056 | -20.348444 |
| *Hoplocryptanthus schwackeanus* | -43.938083 | -20.348417 |
| *Hoplocryptanthus schwackeanus* | -43.954167 | -20.0975 |
| *Hoplocryptanthus schwackeanus* | -43.960556 | -20.09 |
| *Hoplocryptanthus schwackeanus* | -43.963027 | -20.258789 |
| *Hoplocryptanthus schwackeanus* | -43.963056 | -20.258889 |
| *Hoplocryptanthus schwackeanus* | -43.964918 | -20.263007 |
| *Hoplocryptanthus schwackeanus* | -43.965278 | -20.2475 |
| *Hoplocryptanthus schwackeanus* | -43.966667 | -20.098889 |
| *Hoplocryptanthus schwackeanus* | -43.977222 | -20.129722 |
| *Hoplocryptanthus schwackeanus* | -44.199699 | -20.143299 |
| *Hoplocryptanthus schwackeanus* | -44.362167 | -20.121389 |
| *Hoplocryptanthus schwackeanus* | -43.937801 | -19.920799 |
| *Paepalanthus amoenus* | -43.403906 | -20.269189 |
| *Paepalanthus amoenus* | -43.50806 | -20.28778 |
| *Paepalanthus amoenus* | -43.508099 | -20.2875 |
| *Paepalanthus amoenus* | -43.517 | -19.858 |
| *Paepalanthus amoenus* | -43.527778 | -20.265278 |
| *Paepalanthus amoenus* | -43.53 | -20.466111 |
| *Paepalanthus amoenus* | -43.550556 | -20.233056 |
| *Paepalanthus amoenus* | -43.588056 | -20.215556 |
| *Paepalanthus amoenus* | -43.592778 | -20.211944 |
| *Paepalanthus amoenus* | -43.592946 | -20.211927 |
| *Paepalanthus amoenus* | -43.593339 | -20.211081 |
| *Paepalanthus amoenus* | -43.594722 | -20.206389 |
| *Paepalanthus amoenus* | -43.595556 | -20.193333 |
| *Paepalanthus amoenus* | -43.597738 | -20.207469 |
| *Paepalanthus amoenus* | -43.598056 | -20.207778 |
| *Paepalanthus amoenus* | -43.600008 | -20.193203 |
| *Paepalanthus amoenus* | -43.604044 | -20.191902 |
| *Paepalanthus amoenus* | -43.604444 | -20.192222 |
| *Paepalanthus amoenus* | -43.621389 | -20.143611 |
| *Paepalanthus amoenus* | -43.6295 | -20.502556 |
| *Paepalanthus amoenus* | -43.633333 | -20.148056 |
| *Paepalanthus amoenus* | -43.638472 | -20.507722 |
| *Paepalanthus amoenus* | -43.658687 | -20.149842 |
| *Paepalanthus amoenus* | -43.659167 | -20.150278 |
| *Paepalanthus amoenus* | -43.661667 | -20.158056 |
| *Paepalanthus amoenus* | -43.668333 | -20.195833 |
| *Paepalanthus amoenus* | -43.670943 | -20.161718 |
| *Paepalanthus amoenus* | -43.671389 | -20.162222 |
| *Paepalanthus amoenus* | -43.683667 | -20.023067 |
| *Paepalanthus amoenus* | -43.684444 | -20.021389 |
| *Paepalanthus amoenus* | -43.691278 | -20.056667 |
| *Paepalanthus amoenus* | -43.691898 | -20.5208 |
| *Paepalanthus amoenus* | -43.691898 | -20.5208 |
| *Paepalanthus amoenus* | -43.697222 | -20.488333 |
| *Paepalanthus amoenus* | -43.702903 | -20.090481 |
| *Paepalanthus amoenus* | -43.722778 | -20.049167 |
| *Paepalanthus amoenus* | -43.741389 | -20.127778 |
| *Paepalanthus amoenus* | -43.748056 | -20.015278 |
| *Paepalanthus amoenus* | -43.801399 | -20.2533 |
| *Paepalanthus amoenus* | -43.801399 | -20.2533 |
| *Paepalanthus amoenus* | -43.80667 | -19.88667 |
| *Paepalanthus amoenus* | -43.842222 | -20.170833 |
| *Paepalanthus amoenus* | -43.846699 | -19.9856 |
| *Paepalanthus amoenus* | -43.863333 | -20.445556 |
| *Paepalanthus amoenus* | -43.873333 | -20.358611 |
| *Paepalanthus amoenus* | -43.873455 | -20.358528 |
| *Paepalanthus amoenus* | -43.875278 | -20.356389 |
| *Paepalanthus amoenus* | -43.888889 | -20.119167 |
| *Paepalanthus amoenus* | -43.888932 | -20.119164 |
| *Paepalanthus amoenus* | -43.891556 | -20.446913 |
| *Paepalanthus amoenus* | -43.891667 | -20.446944 |
| *Paepalanthus amoenus* | -43.89186 | -20.44698 |
| *Paepalanthus amoenus* | -43.893611 | -20.446389 |
| *Paepalanthus amoenus* | -43.893745 | -20.446443 |
| *Paepalanthus amoenus* | -43.898333 | -20.445278 |
| *Paepalanthus amoenus* | -43.901944 | -20.110556 |
| *Paepalanthus amoenus* | -43.905833 | -20.445833 |
| *Paepalanthus amoenus* | -43.93778 | -19.92083 |
| *Paepalanthus amoenus* | -43.937801 | -19.920799 |
| *Paepalanthus amoenus* | -43.940278 | -20.483333 |
| *Paepalanthus amoenus* | -43.989608 | -20.102775 |
| *Paepalanthus amoenus* | -43.989722 | -20.102778 |
| *Paepalanthus amoenus* | -44.052799 | -20.333099 |
| *Paepalanthus amoenus* | -44.199699 | -20.143299 |
| *Paepalanthus amoenus* | -44.216667 | -20.133333 |
| *Paepalanthus amoenus* | -44.340972 | -20.12 |
| *Paepalanthus magalhaesii* | -43.508099 | -20.2875 |
| *Paepalanthus magalhaesii* | -43.588056 | -20.215556 |
| *Paepalanthus magalhaesii* | -43.651111 | -20.129444 |
| *Paepalanthus magalhaesii* | -43.601667 | -20.191389 |
| *Paepalanthus magalhaesii* | -43.605 | -20.197222 |
| *Paepalanthus magalhaesii* | -43.669444 | -20.211111 |
| *Paepalanthus magalhaesii* | -43.672778 | -20.161389 |
| *Paepalanthus magalhaesii* | -43.673889 | -20.161667 |
| *Paepalanthus magalhaesii* | -43.655556 | -20.126667 |
| *Paepalanthus magalhaesii* | -43.663056 | -20.191111 |
| *Paepalanthus magalhaesii* | -43.664167 | -20.150278 |
| *Paepalanthus magalhaesii* | -43.5925 | -20.173889 |
| *Paepalanthus magalhaesii* | -43.686944 | -20.193333 |
| *Paepalanthus magalhaesii* | -43.687222 | -20.150833 |
| *Paepalanthus magalhaesii* | -44.368889 | -20.125833 |
| *Paepalanthus magalhaesii* | -43.671945 | -20.163056 |
| *Paepalanthus magalhaesii* | -43.668611 | -20.159722 |
| *Paepalanthus magalhaesii* | -43.598333 | -20.2075 |
| *Paepalanthus magalhaesii* | -43.588056 | -20.216111 |
| *Paepalanthus magalhaesii* | -43.603889 | -20.141944 |
| *Paepalanthus magalhaesii* | -43.592778 | -20.211944 |
| *Paepalanthus magalhaesii* | -43.653031 | -20.140413 |
| *Paepalanthus magalhaesii* | -43.671016 | -20.161915 |
| *Paepalanthus magalhaesii* | -43.604537 | -20.191731 |
| *Paepalanthus magalhaesii* | -43.604418 | -20.191592 |
| *Paepalanthus magalhaesii* | -43.604373 | -20.191662 |
| *Paepalanthus magalhaesii* | -43.604569 | -20.191786 |
| *Paepalanthus magalhaesii* | -43.598072 | -20.207117 |
| *Paepalanthus magalhaesii* | -43.668375 | -20.159299 |
| *Paepalanthus magalhaesii* | -43.671541 | -20.162708 |
| *Paepalanthus magalhaesii* | -43.67292 | -20.163777 |
| *Paepalanthus magalhaesii* | -43.592946 | -20.211927 |
| *Paepalanthus magalhaesii* | -43.587984 | -20.215954 |
| *Paepalanthus magalhaesii* | -43.603901 | -20.192033 |
| *Vriesea minarum* | -43.67613 | -19.82237 |
| *Vriesea minarum* | -43.666667 | -19.816667 |
| *Vriesea minarum* | -43.508099 | -20.2875 |
| *Vriesea minarum* | -43.666667 | -19.816667 |
| *Vriesea minarum* | -44.052799 | -20.333099 |
| *Vriesea minarum* | -43.669701 | -19.879999 |
| *Vriesea minarum* | -43.978611 | -20.048611 |
| *Vriesea minarum* | -43.691898 | -20.5208 |
| *Vriesea minarum* | -43.691898 | -20.5208 |
| *Vriesea minarum* | -43.937801 | -19.920799 |
| *Vriesea minarum* | -43.806702 | -19.8864 |
| *Vriesea minarum* | -43.846699 | -19.9856 |
| *Vriesea minarum* | -43.937528 | -20.332361 |
| *Vriesea minarum* | -43.879764 | -20.255969 |
| *Vriesea minarum* | -43.789398 | -20.0875 |
| *Vriesea minarum* | -43.415298 | -19.9594 |
| *Vriesea minarum* | -43.691111 | -20.056667 |
| *Vriesea minarum* | -43.691278 | -20.056667 |
| *Vriesea minarum* | -43.766667 | -19.833333 |
| *Vriesea minarum* | -44.301701 | -20.070299 |
| *Vriesea minarum* | -43.938083 | -20.348444 |
| *Vriesea minarum* | -43.581167 | -20.218806 |
| *Vriesea minarum* | -44.001111 | -20.044722 |
| *Vriesea minarum* | -43.94 | -20.324444 |
| *Vriesea minarum* | -43.683111 | -20.093806 |
| *Vriesea minarum* | -43.687319 | -20.087406 |
| *Vriesea minarum* | -43.868889 | -20.165 |
| *Vriesea minarum* | -43.684722 | -20.062778 |
| *Vriesea minarum* | -43.697222 | -20.488333 |
| *Vriesea minarum* | -43.533333 | -20.435833 |
| *Vriesea minarum* | -43.600278 | -20.052222 |
| *Vriesea minarum* | -43.910278 | -19.961667 |
| *Vriesea minarum* | -43.5775 | -20.018333 |
| *Vriesea minarum* | -43.960556 | -20.09 |
| *Vriesea minarum* | -43.924722 | -20.147222 |
| *Vriesea minarum* | -43.884167 | -20.2725 |
| *Vriesea minarum* | -43.888056 | -20.280556 |
| *Vriesea minarum* | -43.988056 | -20.123056 |
| *Vriesea minarum* | -43.570833 | -20.228056 |
| *Vriesea minarum* | -43.899722 | -20.434444 |
| *Vriesea minarum* | -43.710556 | -20.483333 |
| *Vriesea minarum* | -43.711667 | -20.483889 |
| *Vriesea minarum* | -43.668889 | -19.88 |
| *Vriesea minarum* | -43.693611 | -20.274167 |
| *Vriesea minarum* | -43.650833 | -20.149722 |
| *Vriesea minarum* | -43.582778 | -20.22 |
| *Vriesea minarum* | -43.887222 | -20.279722 |
| *Vriesea minarum* | -43.887778 | -20.281111 |
| *Vriesea minarum* | -44.005 | -20.057222 |
| *Vriesea minarum* | -43.677778 | -19.821945 |
| *Vriesea minarum* | -43.900833 | -20.435 |
| *Vriesea minarum* | -43.977222 | -20.011667 |
| *Vriesea minarum* | -43.862222 | -20.445833 |
| *Vriesea minarum* | -43.901389 | -20.2925 |
| *Vriesea minarum* | -43.965556 | -20.258889 |
| *Vriesea minarum* | -43.899444 | -20.434722 |
| *Vriesea minarum* | -43.856111 | -20.445556 |
| *Vriesea minarum* | -44.009167 | -20.058889 |
| *Vriesea minarum* | -43.887992 | -20.280497 |
| *Vriesea minarum* | -43.650328 | -20.149373 |
| *Vriesea minarum* | -43.582588 | -20.219563 |
| *Vriesea minarum* | -43.582132 | -20.219495 |
| *Vriesea minarum* | -43.886979 | -20.279243 |
| *Vriesea minarum* | -43.887508 | -20.280647 |
| *Vriesea minarum* | -43.886992 | -20.279435 |
| *Vriesea minarum* | -43.881888 | -20.26995 |
| *Vriesea minarum* | -43.678428 | -19.821413 |
| *Vriesea minarum* | -43.684112 | -20.089766 |
| *Vriesea minarum* | -43.676772 | -20.097451 |
| *Vriesea minarum* | -43.671483 | -20.10575 |
| *Vriesea minarum* | -43.670068 | -20.107453 |
| *Vriesea minarum* | -43.668679 | -20.1097 |
| *Vriesea minarum* | -43.6775 | -19.821489 |
| *Vriesea minarum* | -44.004759 | -20.056828 |
| *Vriesea minarum* | -43.899415 | -20.434683 |
| *Vriesea minarum* | -43.901272 | -20.292475 |
| *Vriesea minarum* | -43.977348 | -20.011566 |
| *Vriesea minarum* | -43.711816 | -20.485011 |
| *Vriesea minarum* | -43.697455 | -20.49215 |
| *Vriesea minarum* | -43.525226 | -19.875399 |
| *Vriesea minarum* | -43.862163 | -20.445836 |
| *Vriesea minarum* | -43.900695 | -20.435076 |
| *Vriesea minarum* | -43.965432 | -20.258765 |
| *Vriesea minarum* | -43.540189 | -20.244688 |
| *Vriesea minarum* | -43.683388 | -20.093781 |

Supplementary Table 4. Explained variance of principal components derived from the 19 WorldClim bioclimatic variables used in ENMTML.

| Principal Component | Variance explained (%) | Cumulative variance (%) |
| --- | --- | --- |
| 1 | 53.61 | 53.61 |
| 2 | 29.96 | 83.57 |
| 3 | 9.65 | 93.22 |
| 4 | 3.57 | 96.79 |
| 5 | 2.02 | 98.81 |
| 6 | 0.54 | 99.35 |
| 7 | 0.3 | 99.65 |
| 8 | 0.16 | 99.81 |
| 9 | 0.08 | 99.89 |
| 10 | 0.06 | 99.95 |
| 11 | 0.04 | 99.99 |
| 12 | 0.01 | 100 |
| 13 | 0 | 100 |
| 14 | 0 | 100 |
| 15 | 0 | 100 |
| 16 | 0 | 100 |
| 17 | 0 | 100 |
| 18 | 0 | 100 |
| 19 | 0 | 100 |
|  |  |  |

Supplementary Table 5. Loadings of the 19 WorldClim bioclimatic variables (Bio1–Bio19) on the first four principal components (PC1–PC4) retained for ecological niche modeling in ENMTML. Values represent the correlation coefficients between original variables and each principal component. The first four components cumulatively explained 96.8% of the total variance

| variable | PC1 | PC2 | PC3 | PC4 |
| --- | --- | --- | --- | --- |
| Bio1_QF | -0.3023331 | 0.05504636 | -0.1525334 | 0.10965114 |
| Bio10_QF | -0.3052103 | -0.0104475 | -0.1505982 | 0.09887589 |
| Bio11_QF | -0.2931724 | 0.11220858 | -0.1413642 | 0.14720059 |
| Bio12_QF | 0.22789359 | 0.267254 | -0.1530984 | -0.022343 |
| Bio13_QF | 0.09786365 | 0.32840842 | -0.2202938 | -0.078281 |
| Bio14_QF | 0.18473739 | -0.0849451 | -0.5446891 | 0.17089867 |
| Bio15_QF | -0.1377516 | 0.35618215 | 0.15114029 | -0.0855221 |
| Bio16_QF | 0.14785334 | 0.35889638 | -0.1192459 | -0.044576 |
| Bio17_QF | 0.23517429 | -0.1256648 | -0.4195419 | 0.05852684 |
| Bio18_QF | 0.19938889 | 0.3058916 | 0.02077763 | -0.0652796 |
| Bio19_QF | 0.22929803 | -0.1194078 | -0.4313479 | 0.02469844 |
| Bio2_QF | -0.2050619 | 0.18864557 | -0.2145326 | -0.6106256 |
| Bio3_QF | -0.0258619 | 0.39904172 | -0.0634733 | -0.1305205 |
| Bio4_QF | -0.0782243 | -0.3861184 | -0.0623904 | -0.232803 |
| Bio5_QF | -0.3052538 | -0.006369 | -0.1574099 | 0.05172149 |
| Bio6_QF | -0.2837183 | 0.10986881 | -0.1103113 | 0.35734889 |
| Bio7_QF | -0.2094502 | -0.222771 | -0.1758979 | -0.5478702 |
| Bio8_QF | -0.305026 | 0.01571845 | -0.155003 | 0.08250238 |
| Bio9_QF | -0.2931718 | 0.11220999 | -0.1413721 | 0.14718248 |

Supplementary Table 6. Projected suitable distribution areas (km²) for each species in the Iron Quadrangle under current conditions and future climate scenarios (SSP126 and SSP585 for 2050 and 2090).

| Scenario/ Species distribution (Km^2^) | *Aiouea tetragona* | *Dyckia consimilis* | *Dyckia rariflora* | *Hoplocryptanthus ferrarius* | *Hoplocryptanthus schwackeanus* | *Paepalanthus amoenus* | *Paepalanthus magalhaesii* | *Vriesea minarum* |
| --- | --- | --- | --- | --- | --- | --- | --- | --- |
| Current | 6606.79 | 5712.18 | 3185.75 | 4867.36 | 7856.36 | 7127.18 | 3161.66 | 6419.68 |
| SSP126 2050 | 4324.49 | 6090.42 | 435.26 | 1227.88 | 5468.05 | 4982.20 | 1293.73 | 6184.38 |
| SSP585 2050 | 1398.93 | 1541.08 | 652.09 | 606.31 | 3192.17 | 5162.89 | 258.59 | 4220.89 |
| SSP126 2090 | 2580.24 | 4273.09 | 704.29 | 1431.86 | 4832.02 | 5675.24 | 1211.82 | 6523.27 |
| SSP585 2090 | 0.00 | 338.09 | 0.00 | 0.00 | 40.15 | 92.35 | 3.21 | 718.74 |


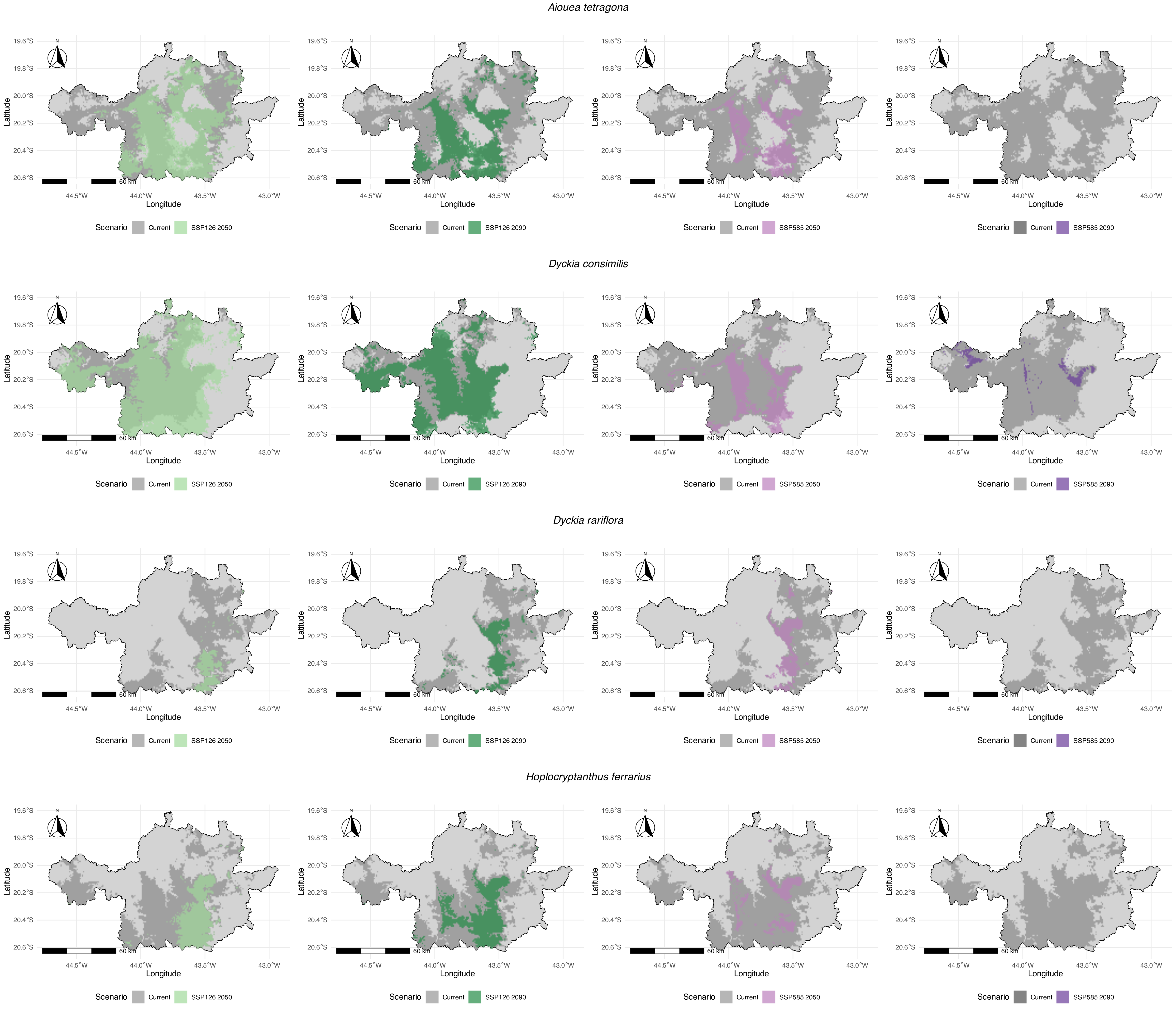


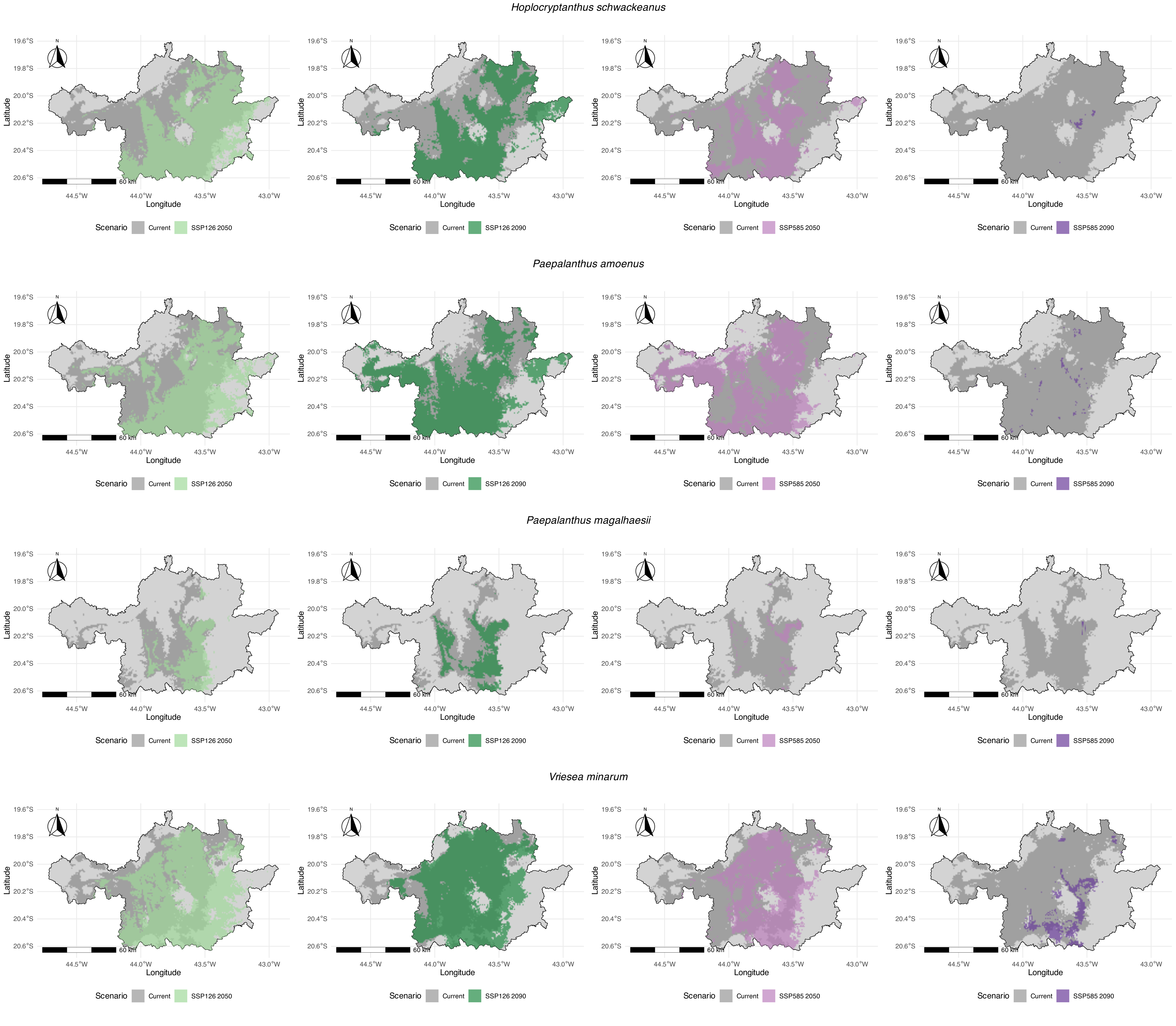


Supplementary Figure 1. **Current and projected distributions of eight plant species in the Iron Quadrangle (IQ) under climate change scenarios.** Each row represents one species (*Aiouea tetragona, Dyckia consimilis, Dyckia rariflora*, Hoplocryptanthus ferrarius, Hoplocryptanthus schwackeanus, Paepalanthus amoenus, Paepalanthus magalhaesii, and Vriesea minarum). Columns show the current distribution (dark grey) overlaid with projections for SSP126 2050 (light green), SSP126 2090 (dark green), SSP585 2050 (light purple), and SSP585 2090 (dark purple). Suitable areas are displayed in binary maps (presence = 1; absence = 0).
